# Functional connectivity for white-tailed deer drives the distribution of tick-borne pathogens in a highly urbanized setting

**DOI:** 10.1007/s10980-025-02101-4

**Published:** 2025-04-22

**Authors:** Marie V. Lilly, Myles Davis, Sara M. Kross, Christopher R. Konowal, Robert Gullery, Sung-Joo Lee, Katherine I. Poulos, Nichar Gregory, Christopher Nagy, Duncan W. Cozens, Doug E. Brackney, Maria del Pilar Fernandez, Maria Diuk-Wasser

**Affiliations:** 1 Department of Ecology, Evolution, and Environmental Biology, Columbia University, New York, NY 10027 USA; 2 NYC Bird Alliance, New York, NY 10010 USA; 3 School of Biological Sciences, University of Canterbury, Christchurch, New Zealand; 4 Department of Entomology, Cornell University, Geneva, NY 14456 USA; 5 EcoHealth Alliance, New York, NY 10018 USA; 6 Mianus River Gorge, Bedford, NY 10506 USA; 7 Department of Entomology, The Connecticut Agricultural Experiment Station, 123 Huntington St, New Haven, CT 06511 USA; 8 Allen School for Global Animal Health, Washington State University, Pullman, WA 99163 USA

**Keywords:** Functional connectivity, Tick-borne hazards, Tick-borne pathogens, Deer occupancy, Urbanization

## Abstract

**Context:**

As cities seek to provide more habitat for wildlife, there may be unintended consequences of increasing tick-borne disease hazards. In the United States, the Northeast is both highly urban and a hotspot for blacklegged ticks (*Ixodes scapularis*) and tick-borne disease emergence. Though tick-borne disease was once considered a suburban and rural problem, tick-borne hazards in urban landscapes are increasing.

**Objectives:**

We hypothesized that multi-scale ecological processes hierarchically contribute to tick-borne hazards across an urbanization gradient. Urban greenspaces with higher functional connectivity to deer movement would have higher deer occupancy at the ‘ecological neighborhood’ scale, resulting in increased blacklegged tick populations and pathogen infection at the scale of within greenspaces.

**Methods:**

To evaluate our hypothesis, we used circuit theory methods to model the impact of functional connectivity on deer occupancy, blacklegged tick abundance, and pathogen infected ticks across an urbanization gradient. We sampled nymphal ticks during their peak activity and deployed wildlife cameras to detect deer at 38 greenspaces across New York City and Long Island, NY from 2022 to 2023.

**Results:**

We found that functional connectivity significantly predicted deer occupancy with cascading effects on abundance of blacklegged nymphal ticks and *Borrelia burgdorferi* infection. We novelly identified a threshold of functional connectivity in urban areas necessary for deer occupancy, tick populations, and tick infection with *B. burgdorferi*, to emerge in urban environments.

**Conclusions:**

We recommend targeted tick-borne hazard mitigation along this functional connectivity threshold as part of urban greenspace management plans. Additionally, we highlight the importance of examining multi-scale landscape drivers of host, tick, and pathogen interactions.

**Supplementary Information:**

The online version contains supplementary material available at 10.1007/s10980-025-02101-4.

## Background

Tick-borne diseases are emerging globally (Rochlin and Toledo 2020). In the United States, tick-borne diseases such as Lyme disease are the most reported vector-borne disease, with more than 400,000 cases a year (Kugeler et al. 2021). The Northeast is the main hotspot for ticks and tick-borne disease emergence in the United States, but there are spatial differences in risk across the region (Diuk-Wasser et al. 2021). In New York (NY) state, blacklegged ticks (*Ixodes scapularis*) are known to transmit several pathogens that cause a great burden to human health, including *Borrelia burgdorferi* (Lyme disease agent)*, Anaplasma phagocytophilum* (Anaplasmosis agent)*,* and *Babesia microti* (Babesiosis agent) (Piedmonte et al. 2018). In their immature life stages, blacklegged ticks are a host generalist ectoparasite that feeds on multiple mammalian, avian, and reptilian hosts (LoGiudice et al. 2003; Ginsberg et al. 2022). Adult blacklegged ticks, however, are host specific, primarily parasitizing white-tailed deer (*Odocoileus virginianus*, hereafter referred to as deer) as their reproductive host (Wilson et al. 1985). Deer are thus considered a keystone host for sustaining blacklegged tick populations (Levi et al. 2012). Both the rise in deer abundance and the reestablishment of deer to peri-urban areas has been associated with the emergence of Lyme disease in North America (Kilpatrick et al. 2017; Eisen and Eisen 2023). In a suburban environment, deer density has been associated with increased tick abundance and local Lyme disease incidence (Kilpatrick et al. 2014). Even in highly urbanized New York City (NYC), connectivity between urban parks has been found to predict blacklegged tick abundance and infection prevalence with *Borrelia burgdorferi* (VanAcker et al. 2019), indicating that urban greenspaces can support deer populations and drive Lyme disease risk (VanAcker et al. 2019, 2023).

Despite extensive studies of urban Lyme disease in Europe (Rizzoli et al. 2014), most studies of tick-borne disease ecology in the US have been limited to rural and suburban areas with relatively little attention towards urban tick-borne disease risk (Kilpatrick et al. 2017). Critical gaps remain in understanding how landscape variables contribute to human disease risk across urbanization gradients (Diuk-Wasser et al. 2021). Increasing wildlife functional connectivity (defined as how a landscape functions as connected from the perspective of a focal species (Pe’er et al. 2011)) in urban areas has been suggested as important for provisioning of ecosystem services, such as urban animal movement and opportunities for human connection to nature (Butler et al. 2022). However, connectivity may also increase tick-borne hazards in highly urban areas, leading to tradeoffs in urban greening strategies (VanAcker et al. 2019; Butler et al. 2022). It is unclear how fine-scale urban landscape patterns, greenspace structure, and tick host occupancy impact tick abundance as well as tick-borne pathogen maintenance and transmission.

**Fig. 1:**
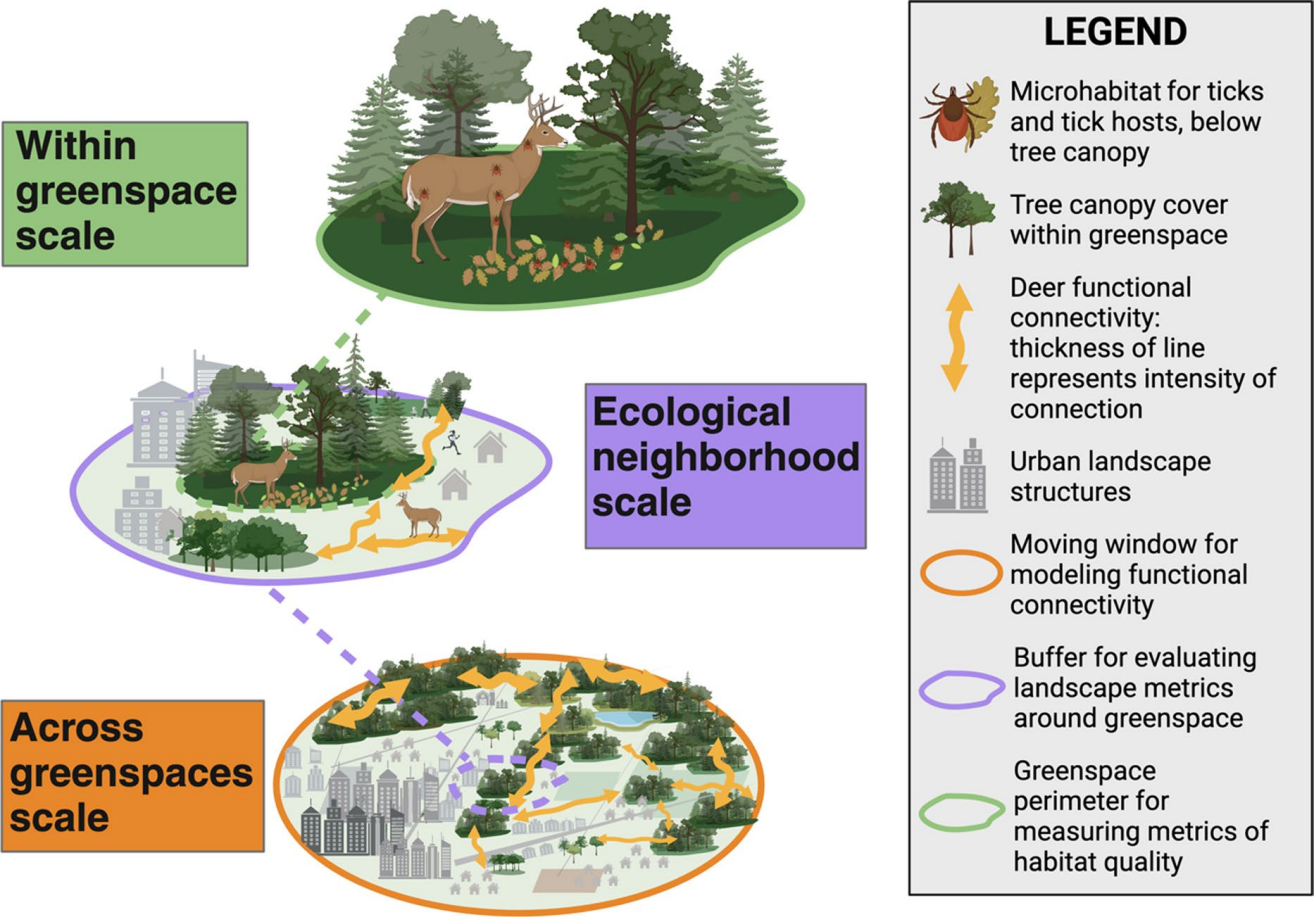
Hypothesized multi-scale ecological processes hierarchically contributing to tick-borne hazard in urban landscapes. Functional connectivity for the keystone tick host (white-tailed deer) across greenspaces determines host occupancy within an ecological neighborhood. Tree canopy cover and microclimate within the greenspace create habitat for tick-borne hazards. Figure created with Biorender.com

The blacklegged tick is dependent on highly vegetated, mostly deciduous forest habitat (Mathisson et al. 2021), mediated by movement of key wildlife hosts, such as deer (Kilpatrick et al. 2017; VanAcker et al. 2023). Habitat patch fragmentation (reduced functional connectivity) has been proposed to increase tick-borne hazards by reducing competition and predation of highly reservoir competent small mammal hosts, most notably the white-footed mouse (*Peromyscus leucopus*) (Ostfeld and Keesing 2000; Schmidt and Ostfeld 2001). Nevertheless, this hypothesis rarely been empirically tested (but see: LoGiudice et al. 2008, Allan et al. 2003, and Diuk-Wasser et al. 2021) and the effect of functional connectivity on tick-borne hazards has not been well examined (but see: VanAcker et al. 2024 and Shaw et al. 2024). In contrast to a proposed positive relationship between fragmentation and tick-borne hazard, we hypothesize that extreme fragmentation will impede tick establishment due to low deer occupancy, leading to a positive connectivity-tick hazard relationship in highly urban areas, and potentially a non-linear relationship across an urbanization gradient. We additionally hypothesize that multi-scale ecological processes hierarchically contribute to tick-borne hazards across an urbanization gradient. Functional connectivity for deer movement across greenspaces is required for deer occupancy at the ‘ecological neighborhood’ (sensu Addicott et al. 1987) scale, leading to increased tick-borne hazards within the habitat patch given suitable microhabitat for tick survival (Fig. 1).

## Methods

### Site selection

Urban greenspaces were selected for wildlife camera trap placement as part of the Urban Wildlife Information Network (UWIN) along a 4 km × 55 km linear transect spanning an urbanization gradient from Staten Island, NYC through Long Island, NY (Magle et al. 2019). Cameras were placed at least 1 km apart to be considered spatially independent for common urban meso-mammals (Magle et al. 2019) and site locations included a mix of city parks (n = 21), cemeteries (n = 5), nature preserves (n = 9), golf courses (n = 6), and public gardens (n = 3) to better capture the distribution of target species sensitive to urban disturbances, such as deer (Gallo et al. 2017). A total of 44 camera placement sites were established at 37 unique greenspaces.

**Fig. 2:**
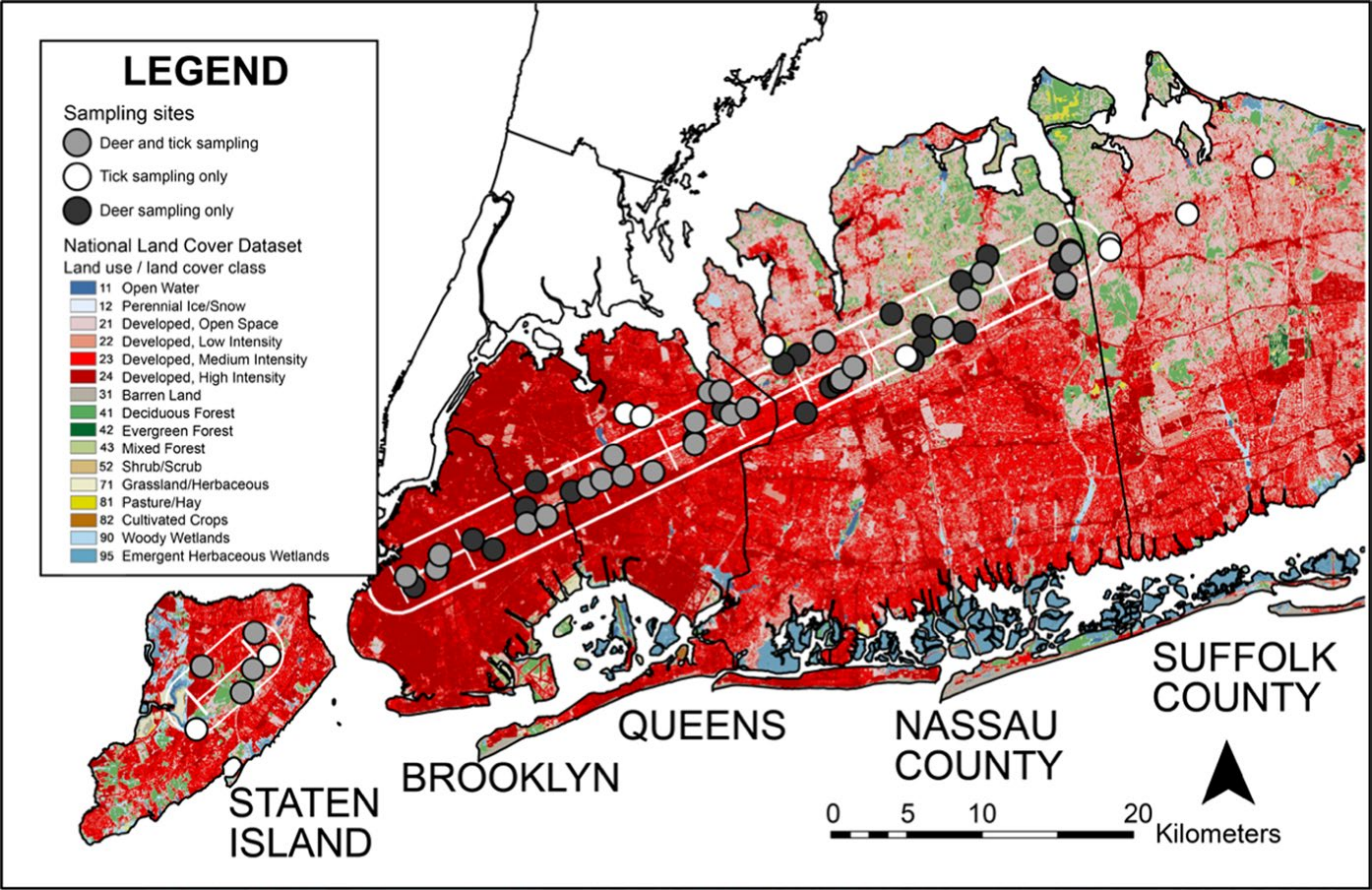
Map of NY study region showing tick and deer sampling sites over National Land Cover Database land use land cover classes (Dewitz and U.S. Geological Survey 2021).

For tick collections, the percent of deciduous or mixed deciduous forest within each greenspace was first calculated from the National Land Cover Database (NLCD) (Dewitz and U.S. Geological Survey 2021). All public use parks, nature preserves, and gardens were filtered to include greenspaces with a patch size of 5 hectares or more and at least 20% forest cover so that tick sampling methods could include 800 m of forested trails (Diuk-Wasser et al. 2006; CDC 2024). After the filtering process, 29 camera trap sites were included for tick sampling. Additional greenspaces along the transect were added for tick collections bringing the total number of sites up to 35 sites in 2022 and 28 sites in 2023, resulting in 38 unique sites sampled over the two-year study period (Fig. 2).

### Wildlife data collection

We deployed one motion-triggered infrared wildlife camera trap (Moultrie M-50 and Browning Strike Force 850) at each site for 28 days in the winter, spring, summer, and fall (four non-consecutive months per year) beginning spring 2022 until Spring 2023. To account for variation in species detection by local habitat type, camera placement within each site was determined using stratified random selection so that an equal proportion of cameras were placed in densely (> 38% tree cover) and lightly forested habitat (< 38% tree cover) within each 5 km segment of the transect (Gallo et al. 2017; Endreny et al. 2020). All photographs were individually tagged to species level and processed using an online database built for UWIN camera trapping research (Magle et al. 2019).

### Nymphal tick collection and pathogen testing

Since tick-borne pathogens are predominantly transmitted to humans via the nymphal tick stage, the density of infected nymphal ticks is widely used as a measure of tick-borne disease hazard (Diuk-Wasser et al. 2012). Thus, we collected blacklegged nymphal ticks twice each year during their peak nymphal activity season in NY (June–July 2022 and 2023). Ticks were collected by drag sampling (CDC 2024) a total of 1600m^2^ (800m^2^ twice per season) within a 500 m radius buffer around a central point, which was the location of the camera trap at UWIN sites and the location of the greenspace centroid in sites without camera traps. Tick sampling locations within each site were selected to include at least 200m^2^ along trail edge, 200m^2^ interior forest (parallel to the trail when possible, at least 10 m away), and 200m^2^ forest-lawn edge with the remaining 200m^2^ based on habitat availability within the 500 m buffer (SI Fig. 1). Each drag was conducted ≥ 10 m apart from each other. Start time, current weather conditions (temperature and percent relative humidity), and GPS coordinates at the start of each transect were noted. The drag cloth was checked every 10 m and all ticks were collected with forceps and placed in ethanol (85% EtOH) or RNA/DNA Shield (Zymo Research, Irvine, CA) vials.

All nymphal blacklegged ticks were tested, up to 100 ticks maximum, for *B. burgdorferi, A. phagocytophilum,* and *B. microti* using a multiplex qPCR. Briefly*,* blacklegged tick nymphs were individually macerated in PBS-G using a mixer mill (251/s) and total nucleic acid extracted using the MagMAX Viral/Pathogen Nucleic Acid Isolation Kit (ThermoFisher, Waltham, MA) and eluted into 50µL. Nucleic acid was subjected to multiplex RT-qPCR targeting three pathogens associated with blacklegged ticks*: B. burgdorferi, B. microti,* and *A. phagocytophilum*. Primer and probe sets were previously validated (Tokarz et al. 2017). The RT-qPCR assay was completed with 4 × TaqPath One-Step Multiplex Mix (ThermoFisher, Waltham, MA) using the following conditions: 2 min. at 25 °C, 10 min. at 53 °C, 2 min. at 95 °C, and 40 cycles of 15 s. of 95 °C and 60 s. of 60 °C. For sites and years where all ticks were tested (n = 50/63), the number of infected nymphs was the total number of infected nymphal ticks. If not all ticks were tested (n = 13/63), the number of infected nymphs was estimated by multiplying the nymphal infection prevalence by the total number of nymphal ticks collected at each site.

### Landscape analyses

#### Land cover layers

We used nationally available land cover and census bureau data in all landscape analysis. SI Table 1 shows complete descriptions of each variable, data source, and justification for use. Briefly here, we modeled functional connectivity using 30 × 30 m resolution land use land cover data from National Land Cover Database (NLCD) (Dewitz and U.S. Geological Survey 2021) and additionally used this dataset for variables of percent water and percent bare soil. We used 30 × 30 m resolution NLCD tree canopy cover (TCC) data and impervious products to calculate percent tree canopy cover and percent impervious surface respectively (Dewitz 2023). We calculated housing density and road density from census bureau TIGER Shapefiles (U.S. Census Bureau 2023a, 2023b). We calculated patch size as the area within a greenspace boundary, derived from shapefiles from the Protected Area Database of the US (U.S. Geological Survey (USGS), 2020), NY State Parks (NYS Parks Administration 2012), and NYC Parks (NYC Parks Open Data Team 2024), or hand drawn.

#### ‘Ecological neighborhoods’: land cover composition surrounding greenspace

We characterized ‘ecological neighborhoods’ by calculating land cover composition from land cover variables (SI Table 1) within buffers of fixed sizes around the greenspace boundaries. We chose buffers around the greenspace perimeter rather than the point location of each camera trap, defining ‘ecological neighborhoods’ as the area surrounding a greenspace of interest in which an ecological process occurs (Addicott et al. 1987; Gregory et al. 2022). We used buffers of 100 m, 500 m, and 1000 m around the greenspace as metrics of accessibility of the greenspace to tick hosts. We chose a 100 m buffer radius because this distance was previously shown to be the best-fit for land cover variable associations with tick density in NYC (VanAcker et al. 2019; Gregory et al. 2022), a 500 m buffer radius because this distance was used in a previous study of deer occupancy in an urban area (Gallo et al. 2019), and a 1000 m buffer radius because this distance encompasses the average urban deer home range in NYC (VanAcker et al. 2023). Tree canopy often correlates with the density of nearby vegetation, so we also assessed tree canopy cover within a 100 m buffer around the camera trap to assess habitat availability of the sampling location within the greenspace and to quantify differences in detection probability for occupancy modeling (Magle et al. 2016).

#### Functional connectivity metrics

We used a circuit theory-based approach to model functional connectivity for deer movement. Functional connectivity was modeled omni-directionally using the Julia package Omniscape.jl (Anantharaman et al. 2020). The omniscape algorithm models current flow across a continuous surface by using a moving window with a user specified radius, block size, and source strength (SI Fig. 2) (McRae et al. 2016). Many traditional connectivity modeling methods, such cost-distance analysis, require designation of core areas and then estimate connectivity only between those areas (Diniz et al. 2020). The nodeless method used by the omniscape algorithm was chosen to better assess potential deer movement at the landscape level across the entire urban landscape including residential and other land use types and to better compare relative connectivity levels across our sites (Phillips et al. 2021).

We reclassified NLCD land cover data at 30 × 30 m resolution to create a resistance surface (Dewitz and U.S. Geological Survey 2021, SI Fig. 3). We used ArcGIS to assign resistance values to each land cover class based on literature data of their permeability to deer movement and gene flow (SI Table 2) (Kelly et al. 2014; Girardet et al. 2015; VanAcker et al. 2019). Each pixel was evaluated as a source for deer movement, with the strength of the source inverse to the level of resistance. Pixels with lower resistance are more likely to serve as a source of animal movement compared to pixels with higher resistance and therefore have higher current flow potential (McRae et al. 2016). We assessed current flow using moving window radii of 100, 200, 300, 400, and 500 pixels (each pixel being 30 × 30 m) to account for variability in maximum deer home range sizes across an urban landscape (VanAcker et al. 2023).

### Model development

#### Deer occupancy models

Camera traps are frequently deployed for studying wildlife (Burton et al. 2015). However, detection of wildlife species on camera is often imperfect (Anderson 2001; Mackenzie et al. 2006), introducing bias into estimations of relative abundance and activity patterns (Tanwar et al. 2021). Occupancy models use a maximum likelihood approach to reduce the issue of imperfect detection (Mackenzie et al. 2006). Occupancy models were therefore chosen to assess patterns of deer distribution and habitat use as they relate to urban landscape variables (Baribeau et al. 2022). Single-season occupancy models were constructed in R using the ‘unmarked’ package (Fiske and Chandler 2011). Single-season occupancy models assume that species occupancy status does not change during the study period and were therefore considered appropriate for our deer sampling effort (Fiske and Chandler 2011). Detection probability *(p*) models were constructed by evaluating seasonality and percent tree canopy within a 100 m buffer of camera trap locations as covariates to account for differences in likelihood of deer detection by each camera and camera trapping season (MacKenzie et al. 2002; Magle et al. 2016). Camera trapping seasons included spring, summer, fall, or winter. Occupancy (*Ψ*) models were then constructed by keeping detection model constant and assessing covariates for occupancy (SI Formula 1).

To identify the best scale at which the mean modeled current flow predicted deer occupancy, we assessed this association at multiple scales of connectivity (moving radii width of 100, 200, 300, 400, and 500 pixels) summarized as mean current flow within buffer sizes (‘ecological neighborhood’ scales) of 100 m, 500 m, and 1000 m around the perimeter of each greenspace as predictors of deer occupancy. We compared the univariate models by using Akaike Information Criterion (AIC) scores and retained the functional connectivity metric and buffer size with the lowest AIC score for future analyses (Burnham and Anderson 2002; VanAcker et al. 2019). All other landscape variables were similarly assessed at buffer sizes of 100 m, 500 m, and 1000 m as predictors of deer occupancy. Multivariate models were next constructed to include landscape variables within the buffer size that performed best in univariate models. All covariates were scaled to compare effect sizes and covariates with a Pearson's correlation coefficient > 0.5 were not included in the same model to reduce multicollinearity (SI Fig. 4). Functional connectivity, housing density, percent tree canopy, percent impervious surface, and road density were all collinear and therefore separately assessed by building five global models with each of these covariates and then using the dredge function to test every combination of additional model covariates (percent water, patch size, and human detection frequency) from each global model until the top candidate models were determined (MacKenzie et al. 2002; MacKenzie and Bailey 2004). Model fit was assessed by comparing AIC scores (Burnham and Anderson 2002). The variance inflation factor (VIF) was also calculated for all covariates in the fitted model parameters. For models with a VIF of > 5, covariates were removed and analyzed separately.

A Mackenzie-Bailey goodness of fit test for single-season occupancy model was also performed for each top candidate model and model averaging was used for the top three candidate models within < 2 AIC units to predict occupancy probabilities at each site (MacKenzie and Bailey 2004). Occupancy probabilities at each site were also calculated for each of the top three candidate models individually.

#### Tick hazard models

We used negative binomial Generalized Linear Mixed Models (GLMMs) with site and year as random effects to evaluate predictors of nymphal tick abundance and number of nymphal ticks infected with *B. burgdorferi, A. phagocytophilum,* and *B. microti* across the urbanization gradient (see supplemental Section S1 for additional details on model construction). Landscape variables and the best-fit modeled deer occupancy were assessed as predictors using bidirectional stepwise procedure and the best-fit model was selected by comparing AIC scores (Burnham and Anderson 2002). Total meters dragged was included as an offset in each model and predictor variables with a Pearson's correlation coefficient > 0.5 were not included in the same model (SI Fig. 5). Moran’s I was used to test for spatial autocorrelation of the residuals.

## Results

We collected field data from 2022–2023 resulting in 4928 camera trapping days capturing 4,690 photos of deer across 44 sites and the collection of 3026 nymphal blacklegged ticks across 38 sites. Overall nymphal infection prevalence with *B. burgdorferi* was 28.67%, with *B. microti* was 14.48%, and with *A. phagocytophilum* was 3.35%. Out of 38 tick collection sites, nymphal blacklegged ticks were found at 27 sites (SI Table 3). Across four seasons at 44 camera stations, deer were captured on camera at 19 sites.

### Deer—detection models (p)

With 44 cameras operating for four seasons (4 weeks per season), there were 704 possible data points which were summarized as presence or absence of deer within 1 week (Magle et al. 2016). Analyses of detection probability (*p*) found that the best-fit model for detection probability included season (spring, summer, fall, or winter) and percent tree canopy cover (TCC) within a 100 m buffer of camera trap coordinates as covariates (SI Table 4). There was a significant positive association (estimate = 0.76, p < 0.001) between percent TCC and probability of deer detection as well as significant negative differences between the fall, spring, and summer seasons compared to winter season and probability of deer detection (Table 1). Winter was the only season found to have a significant effect on probability of deer detection (SI Table 5).

**Table 1:** Best-fit model for probability of detection (*p*) of deer

Model component	Estimate	SE	z	P( >|z|)
(Intercept)	− 0.73	0.28	− 2.62	0.009
TCC	0.76	0.22	3.51	< 0.001
Fall	− 1.03	0.38	− 2.69	0.007
Spring	− 1.27	0.46	− 2.78	0.005
Summer	− 0.93	0.37	− 2.50	0.013

TCC = Percent tree canopy cover within 100 m of camera trap

### Deer—occupancy models (Ψ)

Functional connectivity estimated at all moving window radii and landscape metrics at all buffer distances were highly correlated (> 0.9) and mean functional connectivity calculated from a moving window radius of 300 pixels (corresponding to 9,000m^2^) within a buffer size of 1000 m around the greenspace perimeter yielded the best model fit for predicting deer occupancy. With the detection model held constant, analysis of univariate models to select buffer size for all other landscape variables resulted in a buffer size of 1000 m as the best-fit for percent impervious surface, percent tree canopy cover, and housing unit density. A buffer size of 500 m was the best-fit for road density, and a buffer size of 100 m was the best-fit for percent water.

**Fig. 3:**
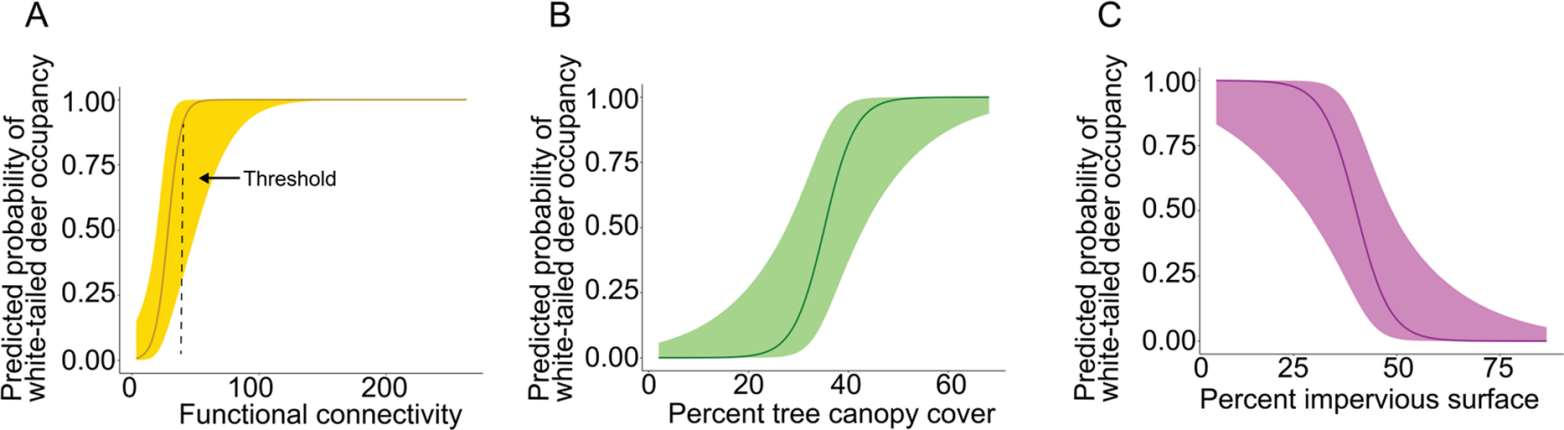
Relationship between predicted white-tailed deer occupancy probability and (A) Functional connectivity, (B) Percent tree canopy cover, and (C) Percent impervious surface. All landscape variables calculated within a 1000 m buffer around each site. Landscape variables were unscaled for figure construction

Five separate multivariate global models were built to avoid collinearity between functional connectivity, housing density, percent tree canopy, percent impervious surface, and road density. Human detection frequency, percent water, and patch size were included in each global model since Pearson's correlation coefficient was < 0.5 between these variables and all other variables assessed. Model dredging was then used to identify candidate models and covariates were removed from models with variance inflation factors of > 5, resulting in three top candidate occupancy models within 2 AIC units of each other. These top candidate occupancy models each had one covariate and included (1) percent tree canopy cover, (2) percent impervious surface, and (3) mean functional connectivity (SI Table 6). Functional connectivity had the largest effect size on deer occupancy with a significant (p < 0.01) positive estimate of 11.09, followed by percent tree canopy cover with a significant (p < 0.01) positive estimate of 6.15, and percent impervious surface with a significant (p < 0.01) negative estimate of − 5.85 (Table 2, Fig. 3). Scaling the covariates allowed us to directly compare model estimates and determine relative effect on deer occupancy, but covariates were unscaled in figure construction to increase interpretability (Fig. 3).

**Table 2:** Estimates for top predictors of deer occupancy with covariates scaled to compare effect sizes

Model component	Estimate	SE	z	P( >|z|)
(Intercept)	3.04	1.79	1.70	0.09
Functional connectivity	11.09	4.08	2.72	< 0.01
(Intercept)	− 1.53	1.04	− 1.48	0.14
TCC	6.15	2.27	2.70	< 0.01
(Intercept)	− 1.26	0.91	− 1.39	0.16
Percent impervious surface	− 5.85	2.24	− 2.62	< 0.01

TCC = Percent Tree Canopy Cover

### Tick hazard

Occupancy estimates from the top three candidate deer occupancy models as well as the model averaged occupancy estimate were evaluated as univariate predictors of nymphal blacklegged tick abundance. For sites where both deer and tick data were collected (n = 29/38), the modeled deer occupancy value was used as a covariate. For sites where tick data was collected but there was no camera (n = 9/38), the predicted deer occupancy value was used as a covariate. The functional connectivity deer occupancy model outperformed all other occupancy models as a predictor of nymphal blacklegged tick abundance and is hereafter referred to as “deer occupancy” (SI Table 7).

**Fig. 4:**
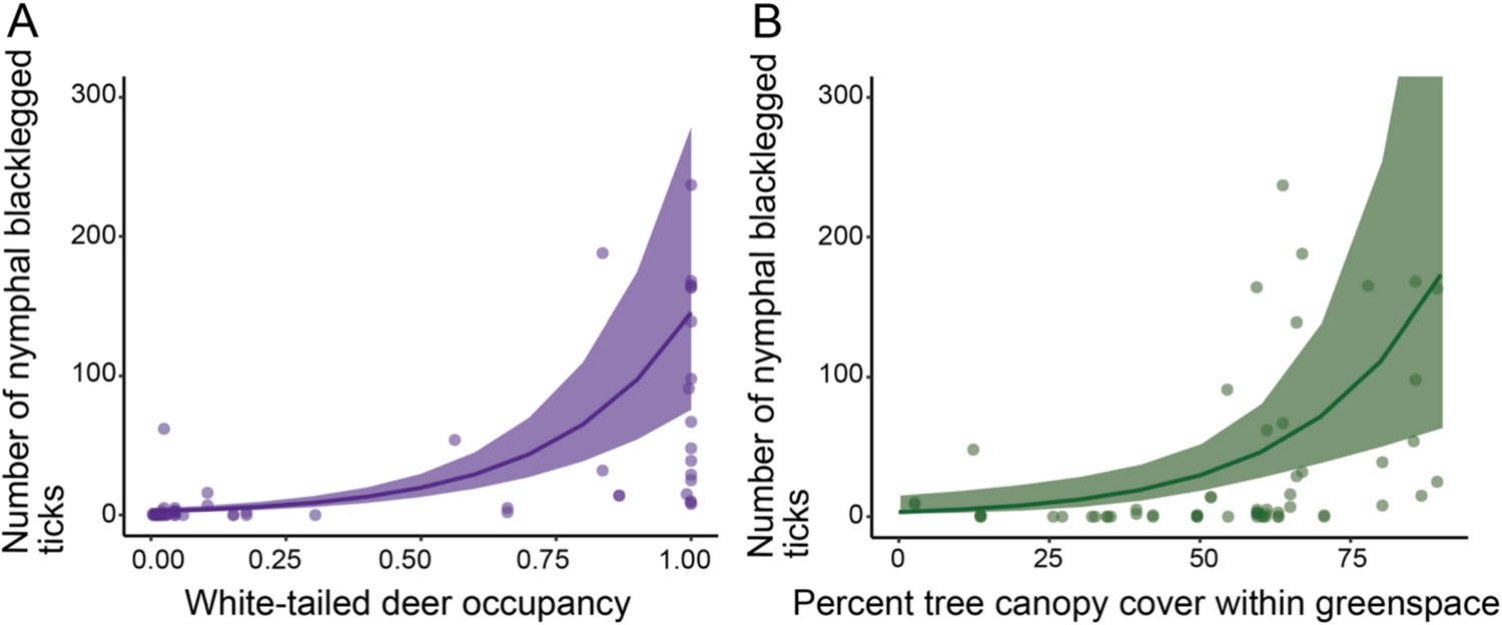
Predicted number of nymphal blacklegged as (A) a function of white-tailed deer occupancy with a purple regression line and standard error (B) a function of percent tree canopy cover within the greenspace with a green regression line and standard error. For both plots, plot points indicate field collected numbers of ticks in 2022–2023 per 1600m^2^ of sampling. Y-axis limited to < 300 ticks for visualization

**Fig. 5:**
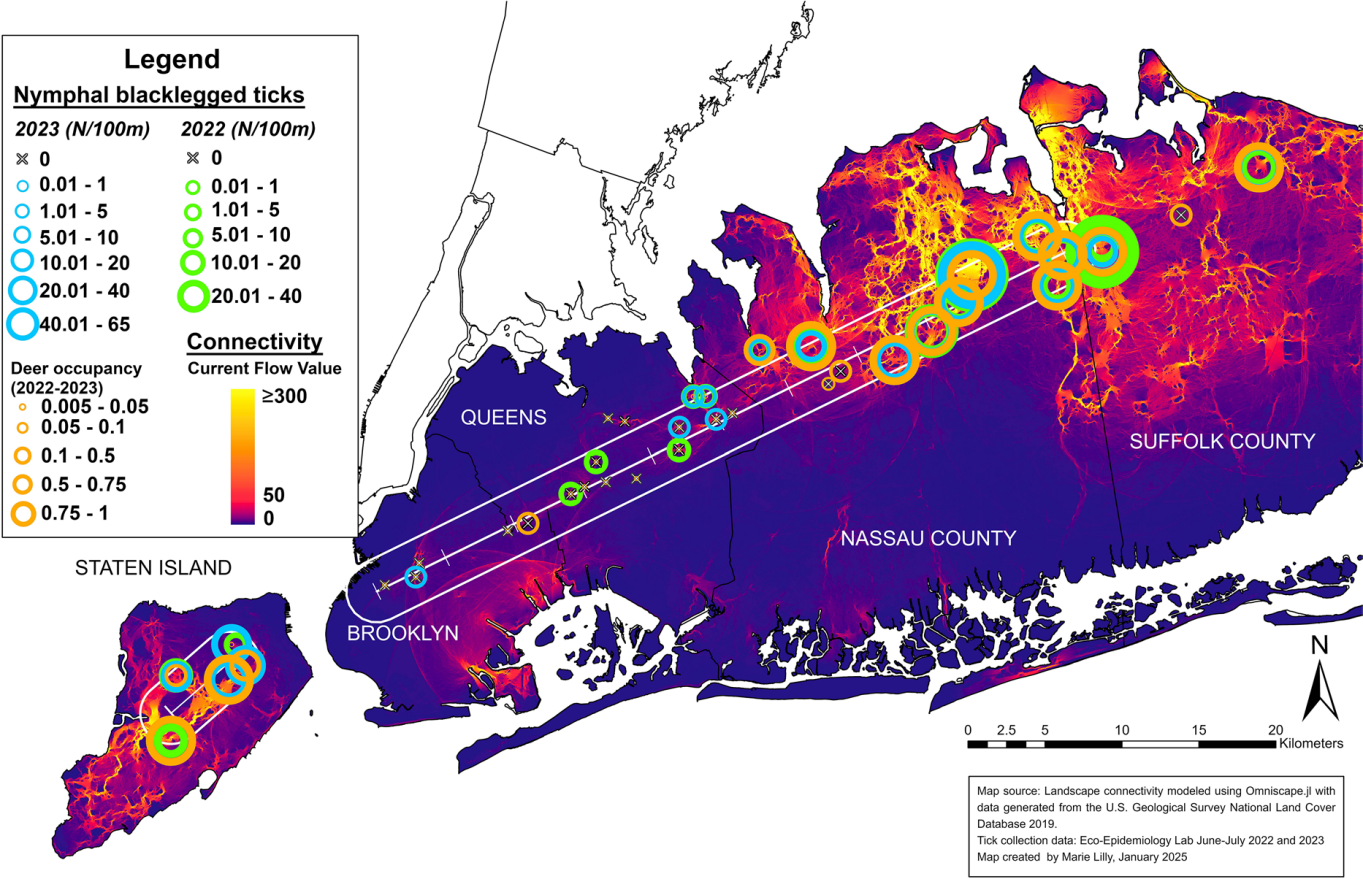
Nymphal blacklegged tick collections standardized to ticks/100m^2^ from 2022 and 2023 along with modeled white-tailed deer occupancy using camera trap data from April 2022, July 2022, October 2022, and January 2023. Deer and tick data shown with UWIN transect and over modeled functional connectivity to deer movement as relative current flow

We found deer occupancy and percent tree canopy cover within the greenspace were both included in the best-fit model for predictors of nymphal blacklegged tick abundance, and both were significantly positively associated with nymphal blacklegged tick abundance (Table 3, Fig. 4). Two other candidate models were within 2 AIC units of the top model, and included deer occupancy, percent tree canopy cover within the greenspace, and patch size or percent bare soil within a 100 m buffer around the greenspace perimeter. Both latter additional predictor variables showed a slight positive effect, but neither were significant. Functional connectivity alone was not included in the best-fit model but was also a significant positive predictor of nymphal tick abundance (Fig. 5).

**Table 3:** Best-fit negative binomial generalized linear mixed effects model assessing nymphal blacklegged tick abundance as a function of best-fit deer occupancy and percent tree canopy cover within the greenspace. An offset was used for total distance dragged and site and year were included as random effects

Model component	Estimate	SE	z value	Pr( >|z|)
(Intercept)	− 9.04	0.81	− 11.17	< 0.001
Deer occupancy	4.43	0.57	7.79	< 0.001
Within greenspace TCC	0.03	0.01	2.33	0.02

TCC = percent tree canopy cover

**Fig. 6:**
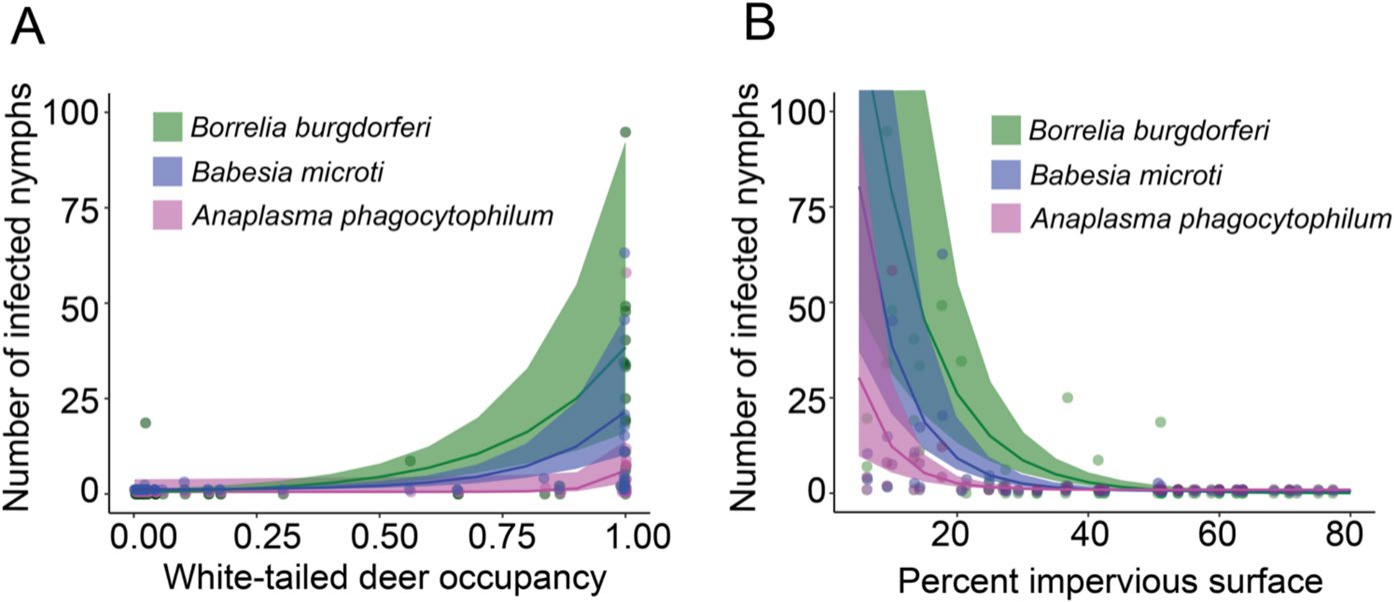
Predicted number of infected nymphal blacklegged ticks with *Borrelia burgdorferi*, *Anaplasma phagocytophylum* and *Babesia microti* predicted by (A) white-tailed deer occupancy*;* (B) percent impervious surface. Points represent field data collections per 1600m^2^ and trendline with standard error represents model prediction. Y-axis limited to < 100 infected ticks for visualization

Following similar analyses, the number of nymphs infected with *B. burgdorferi* was best predicted by deer occupancy (a positive relationship) while both number of nymphs infected with *B. microti* and number of nymphs infected with *A. phagocytophilum* were best predicted by percent impervious surface within a 1000 m buffer of the greenspace (a negative relationship). Deer occupancy also significantly (Estimate = 5.70, p < 0.001) predicted nymphs infected with *B. microti* and strongly (Estimate = 17.36, p = 0.06) predicted *A. phagocytophilum*, though deer occupancy was not included in the best-fit model (Table 4, Fig. 6, SI Fig. 6).

**Table 4:** Generalized linear mixed effect models showing number of nymphal blacklegged ticks infected with (1) *Borrelia burgdorferi*, (2) *Babesia microti*, and (3) *Anaplasma phagocytophilum* as functions of deer occupancy and impervious surface predictor variables. All models were constructed using a zero-inflated negative binomial family. An offset was used for total distance dragged and site and year were included as random effects

Response variable	Model component	Estimate	SE	z value	Pr( >|z|)
*Borrelia burgdorferi*	(Intercept)	− 10.58	1.01	− 10.49	< 0.001
	Deer occupancy*	5.88	1.05	5.58	< 0.001
*Borrelia burgdorferi*	(Intercept)	− 2.34	0.99	− 2.36	0.02
	Percent impervious surface	− 0.14	0.02	− 5.57	< 0.001
*Babesia microti*	(Intercept)	− 3.23	0.83	− 3.91	< 0.001
	Percent impervious surface*	− 0.14	0.03	− 5.55	< 0.001
*Babesia microti*	(Intercept)	− 11.58	1.16	− 9.99	< 0.001
	Deer occupancy	5.70	1.20	4.75	< 0.001
*Anaplasma phagocytophilum*	(Intercept)	− 3.01	0.92	− 3.27	0.001
	Percent impervious surface*	− 0.19	0.04	− 4.37	< 0.001
*Anaplasma phagocytophilum*	(Intercept)	− 23.30	9.11	− 2.56	0.01
	Deer occupancy	17.36	9.27	1.87	0.06

^*^Best-fit model

## Discussion

As cities strive to establish, augment, and connect green spaces, it is increasingly important to understand the impact of such practices on wildlife and associated hazards. This study identifies, for the first time, a direct association between functional connectivity, deer occupancy, and density of tick populations in urbanized landscapes. We found that urban landscape composition and configuration across an urbanization gradient drives deer occupancy, the keystone wildlife host species for blacklegged tick establishment (Wilson et al. 1985; Levi et al. 2012). Functional connectivity had a cascading effect on tick numbers and *B. burgdorferi* (causative agent of Lyme disease) infection status mediated by deer occupancy, supporting our hypothesis that functional connectivity facilitating deer movement drives deer occupancy and mediates nymphal blacklegged tick abundance and pathogen infection (Fig. 1, SI Fig. 6).

We used novel applications of a method for modeling connectivity (McRae et al. 2016) and found that our functional connectivity metric had the largest effect size, of all variables assessed, on deer occupancy, with a threshold of connectivity indicated before modeled occupancy reaches an asymptote of total (1) occupancy. Other landscape variables of percent tree canopy cover and percent impervious surface reveal a nearly perfect inverse relationship with each other with percent tree canopy cover increase showing a positive effect on deer occupancy and percent impervious surface showing a negative effect on deer occupancy. Our results identify thresholds of surrounding functional connectivity, percent tree canopy cover, and percent impervious surface required for the establishment of deer populations in highly urban greenspaces.

Functional connectivity, impervious surface, and tree canopy cover were highly correlated with each other (SI Fig. 3) and can all be used as metrics of urbanization, though there are important differences in the information they contain as it regards to deer occupancy (Wan et al. 2023). The functional connectivity metric accounts for both habitat patch size and surrounding habitat configuration, while percent tree canopy cover and percent impervious surface are simpler metrics that only consider habitat composition in the buffer area considered (Anantharaman et al. 2020; Diniz et al. 2020). Our best functional connectivity model was based on a moving window radius of 300 pixels (9,000m^2^) which corresponds to the scale of the maximum urban deer home range size (10,490m^2^) detected through GPS tracking on NYC’s Staten Island Borough, NY (VanAcker et al. 2023). Connectivity across 9–10,000m^2^ would be required for deer to utilize their maximum home range. VanAcker et al. (2023) finding that the average urban deer home range size is 500–1700m^2^ is additionally consistent with our ecological neighborhood buffer size of 1000m^2^ around each greenspace which lead to the most informative scale of landscape variables to predict deer occupancy (VanAcker et al. 2023).

In addition to deer occupancy, the deer detection probability model included a positive association between deer detection and the winter season and a positive association between deer detection and percent tree canopy cover within a 100 m buffer around the camera trap, suggesting that local tree canopy cover may be an indicator of deer space use within a greenspace rather than understory brush height and therefore where a camera may be more likely to detect deer. Although neither human detection frequency nor housing density were included in the top candidate models for deer occupancy, it is noteworthy that there was a significant negative association with deer occupancy and both variables, suggesting a potential avoidance of high human activity which aligns with a previous study of deer habitat use across urban landscapes (Magle et al. 2014).

Occupancy models are powerful tools for quantifying wildlife distributions (Gould et al. 2019) and making conservation decisions (MacKenzie and Reardon 2013) but have rarely been applied to pathogen and vector host population distributions in disease ecology research (but see: Adams et al. 2010; Estrada-Peña and de la Fuente 2016). We innovatively used occupancy models to identify a direct association between deer and increasing tick populations across an urbanization gradient. Deer density has been directly linked to blacklegged tick abundance in rural and suburban areas (Wilson et al. 1985; Kilpatrick et al. 2014), but less research has been conducted in urban areas and the shape of the association has rarely been assessed (but see: VanAcker et al. 2019, 2024; Bastard et al. 2024). While deer occupancy was the strongest predictor of nymphal blacklegged tick abundance, landscape configuration variables without wildlife host data were also significant predictors of nymphal blacklegged abundance, though not included in the top candidate model. In situations where wildlife host data is not available or impractical to collect, functional connectivity along with other landscape features could serve as a proxy for deer occupancy in tick hazard models. Although we did not evaluate the effect of additional wildlife hosts on tick abundance, the landscape variables evaluated in this study can be broadly used as proxies of habitat quality and movement potential for several other blacklegged tick hosts, such as the white-footed mouse which most commonly inhabits woodland areas with high densities of trees and shrubs (Kaufman et al. 1985; Munshi-South 2012).

Although deer are a non-competent host for *B. burgdorferi* (Telford et al. 1988)*,* we found a significant positive association between deer occupancy and number of nymphal ticks infected with *B. burgdorferi*, which is consistent with previous studies (Kilpatrick et al. 2014; VanAcker et al. 2019). We thus refer to deer as keystone (tick amplifier) host and their impact on pathogen force of infection (*R*_0_ > 1) mediated by increased tick abundance as a ‘cascading’ effect on the tick-host–pathogen transmission cycle (Tufts et al. 2023). Studies of white-footed mice in urban settings indicate that this key reservoir host for *B. burgdorferi* is abundant and therefore likely not limiting (Munshi-South 2012), making deer-mediated tick amplification the key determinant of pathogen persistence.

Regarding the other tick-borne pathogens, *B. burgdorferi* has higher transmission efficiency than *B. microti* or A*. phagocytophilum* (Mather et al. 1990; Perez et al. 2020), with *B. burgdorferi* able to persist at lower tick densities (Tufts et al. 2023). With a lower force of infection, *B. microti* and *A. phagocytophilum* may require higher tick densities for establishment and therefore be more strongly affected by urban landscape composition and configuration (Piedmonte et al. 2018; Perez et al. 2020; Tufts et al. 2023), which is supported by our finding that *B. microti* and *A. phagocytophilum* were negatively associated with percent impervious surface around the greenspace. While we evaluated landscape variables and occupancy of deer, a keystone tick amplifying host, we did not evaluate pathogen reservoir infection status. We noted an unusually high infection prevalence with *B. microti* (14.48%) across all sites and years (Diuk-Wasser et al. 2014), with four sites exhibiting a higher infection prevalence with *B. microti* than with *B. burgdorferi.* This high *B. microti* infection prevalence could be caused by coinfection enhancement of *B. microti* by *B. burgdorferi* (Dunn et al. 2014) or could be explained by large populations of white-footed mice, which are the primary reservoir host for *B. microti* (Tufts and Diuk-Wasser 2021)*.* Large white-footed mouse population abundances would disproportionately impact *B. microti* persistence since *B. burgdorferi* is a host generalist pathogen (Combs et al. 2023). White-footed mice have also been found to vertically transmit *B. microti* to their offspring (Tufts and Diuk-Wasser 2021), further increasing *B. microti* persistence in reservoir hosts and thus the likelihood of tick infection (Tufts et al. 2023)*.* Future studies should examine reservoir host community infection dynamics to fully understand drivers of tick-borne pathogen emergence across urbanization gradients (Tufts et al. 2023; Bastard et al. 2024).

Our finding of a significant positive association between nymphal blacklegged tick abundance and percent tree canopy cover within an urban greenspace could be explained by tick microhabitat quality. In addition to requiring wildlife hosts to complete their life cycle, blacklegged ticks require humid conditions for survival. Blacklegged ticks are highly sensitive to desiccation and seek out microclimates with higher ambient humidity, such as such as leaf litter and low vegetation (Knülle and Rudolph 1982). Increased tree canopy cover has been shown to increase soil moisture (Greiser et al. 2024) and therefore may lead to better tick microhabitat quality in urban greenspaces with a larger proportion of tree canopy cover. Although habitat patch size has been found to be important in urban tick population and pathogen infection maintenance (Shaw et al. 2024), habitat patch size was not included in our best-fit models for tick or pathogen abundance. Our habitat patch size metric was measured using management boundaries and our sampling methods excluded habitat patches smaller than five hectares, which may have limited the effect of patch size on our results.

Maintaining and increasing functional connectivity has become a priority in many wildlife conservation and landscape management plans across the globe (Hilty et al. 2020). Functional connectivity has many benefits for wildlife mobility and improving conservation efforts of endangered or rare megafauna in human modified landscapes (Schoen et al. 2022; Suraci et al. 2023). Cities like NYC are embracing green infrastructure and enhancement of greenspace connectivity as methods to combat negative impacts of climate change (Hansen et al. 2015; Zhang et al. 2019). There are also cultural and social benefits to greenspace connectivity via green corridors and greenways, such as higher social connectivity (Butler et al. 2022), increased quality of life (Shafer et al. 2000), and improved physical and mental health (Larson et al. 2016; Wang et al. 2022). While there are many benefits of greenspace connectivity in urban areas, it is important for park managers, city planners, and local government officials to also consider the potential hazards that can result from increased functional connectivity in urban greenspace design. To balance maintenance of greenspace connectivity along with hazard reduction, we suggest targeted deer management and tick hazard mitigation strategies in areas with increased functional connectivity, such as at the connectivity threshold identified in this study.

## Conclusion

Our findings highlight the importance of examining landscape structure and composition at multiple scales in evaluating drivers of host, tick, and pathogen interactions especially in heterogeneous urban environments undergoing changes to surrounding land cover and tick-borne pathogen emergence. Through this study we identified functional connectivity as the strongest predictor of deer occupancy across an urbanization gradient and deer occupancy as the strongest predictor of both nymphal tick abundance and number of *B. burgdorferi* infected nymphal ticks. As urban areas continue to grow and regreening strategies are embraced, it is important to recognize the potential tick-borne hazards associated with regreening strategies and adapt accordingly.

## Supplementary Information

Below is the link to the electronic supplementary material.

Supplementary file1 (DOCX 5397 KB)

## Data Availability

The data presented in this study are contained within the article and supplementary information or are deposited in the github repository “m-lilly/connectivity_deer_ticks.”
