# Supplementary material for: Functional connectivity for white-tailed deer drives the distribution of tick-borne pathogens in a highly urbanized setting
Source: Landsc Ecol. 2025 Apr 22;40(5):87. doi: 10.1007/s10980-025-02101-4 (PMC12011924; doi:10.1007/s10980-025-02101-4)
Supplement: Supplementary file 1 — Supplementary file1 (DOCX 5397 KB) [file 10980_2025_2101_MOESM1_ESM.docx]

***Landscape Ecology***

**Functional connectivity for white-tailed deer drives the distribution of tick-borne pathogens in a highly urbanized setting**

Marie V. Lilly, Myles Davis, Sara M. Kross, Christopher R. Konowal, Robert Gullery, Sung-Joo Lee, Katherine I. Poulos, Nichar Gregory, Christopher Nagy, Duncan W. Cozens, Doug E. Brackney, Maria del Pilar Fernandez, Maria Diuk-Wasser

**Supplementary Information**


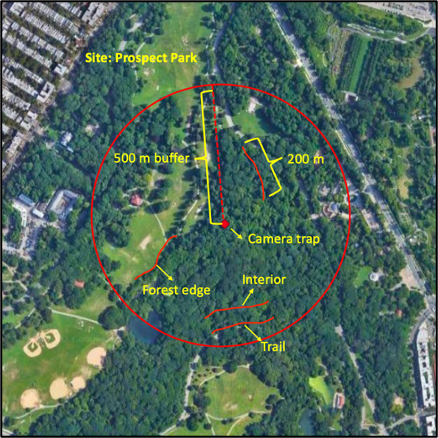


**SI Figure 1**. Example of field site setup with camera trap as the central point and tick drag transect types within a 500m buffer around the camera.


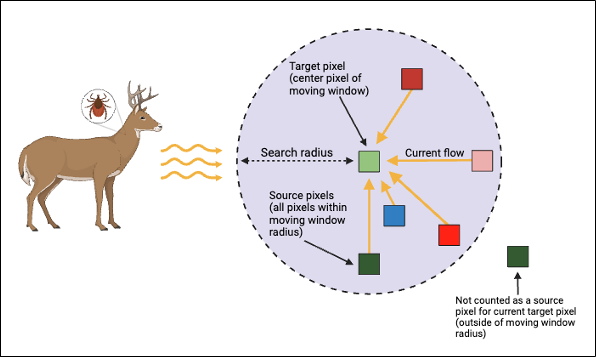


**SI Figure 2.** Illustration of the moving window omniscape algorithm measuring current flow as a metric of functional connectivity to deer movement and tick population establishment. Figure adapted from McRae et al. 2016.

**
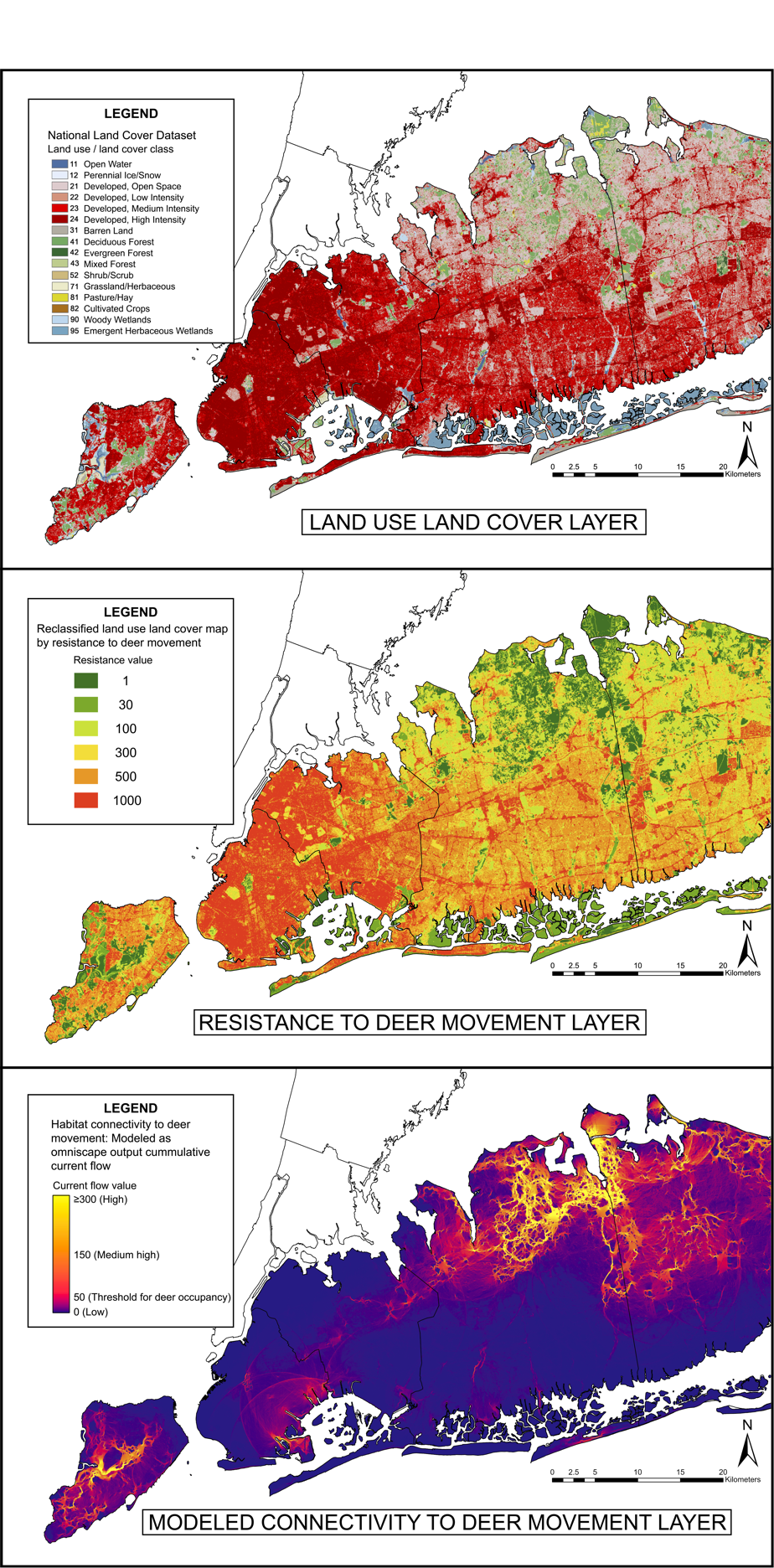
**

**SI Figure 3.** Spatial layers for modeled functional connectivity. Land use land cover layer, derived resistance layer, and modeled output of connectivity to deer movement layer.


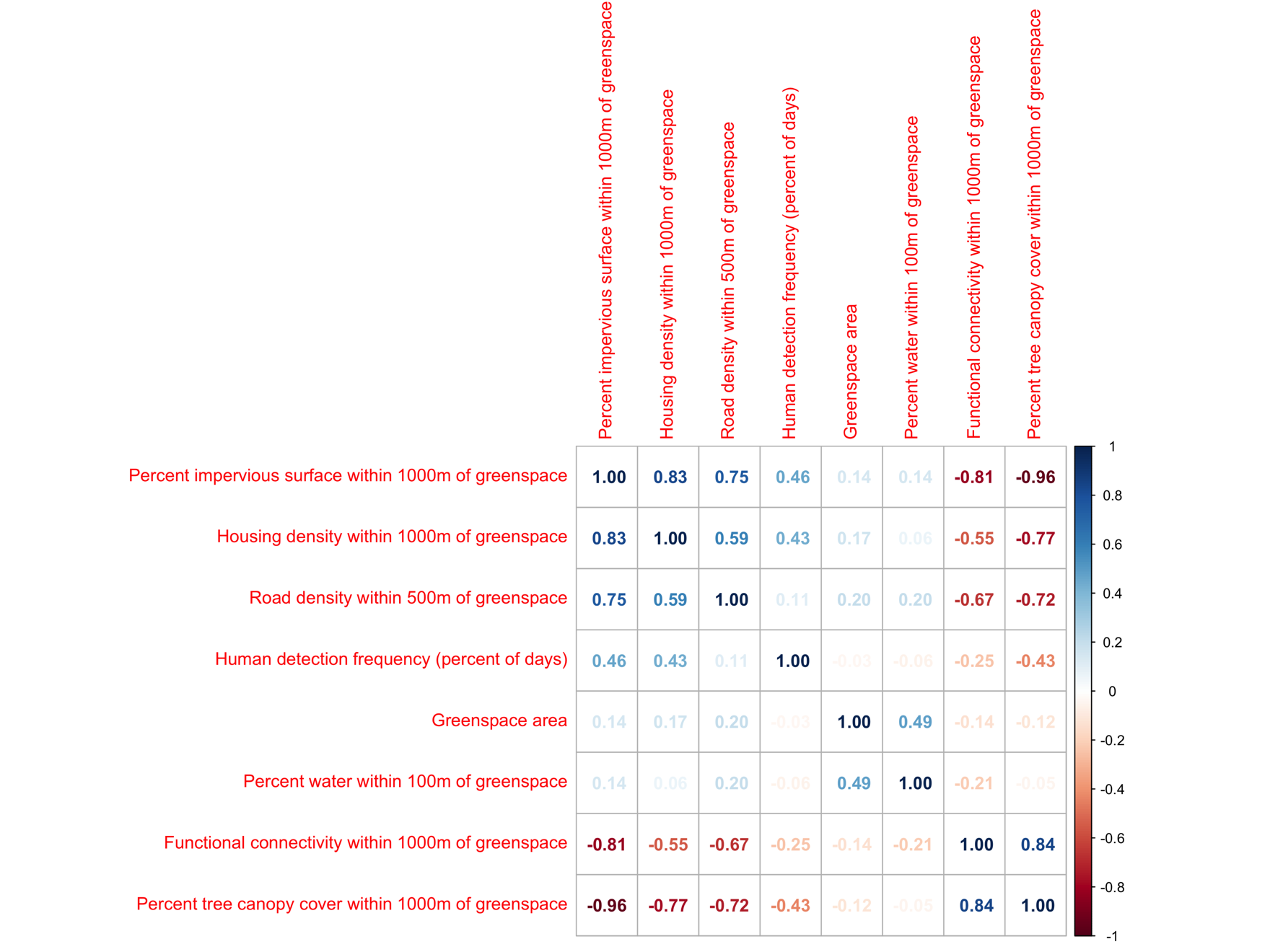


**SI Figure 4.** Correlation matrix of covariates assessed in deer occupancy model.

**
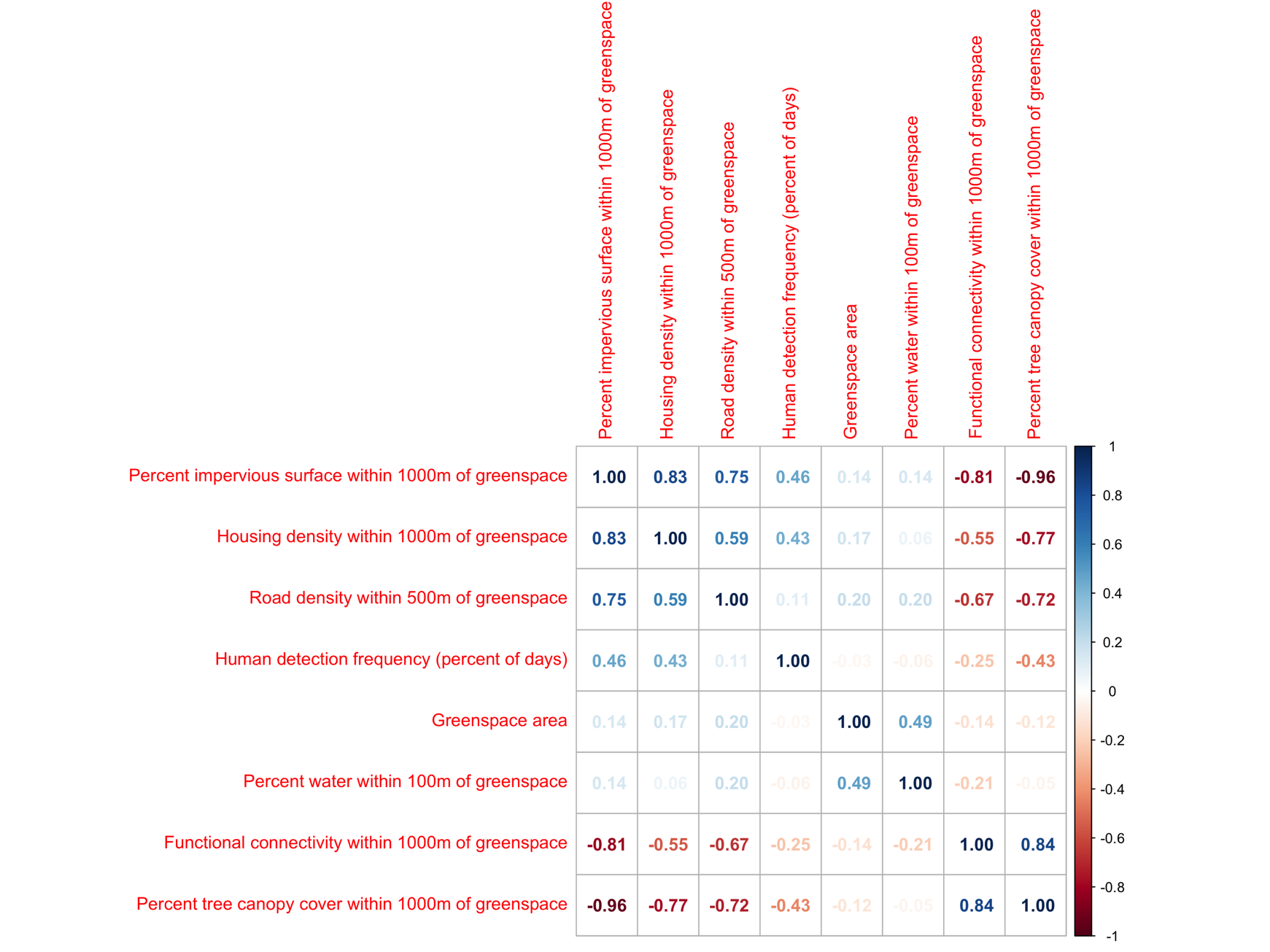
**

**SI Figure 5.** Correlation matrix of variables assessed in tick hazard model.


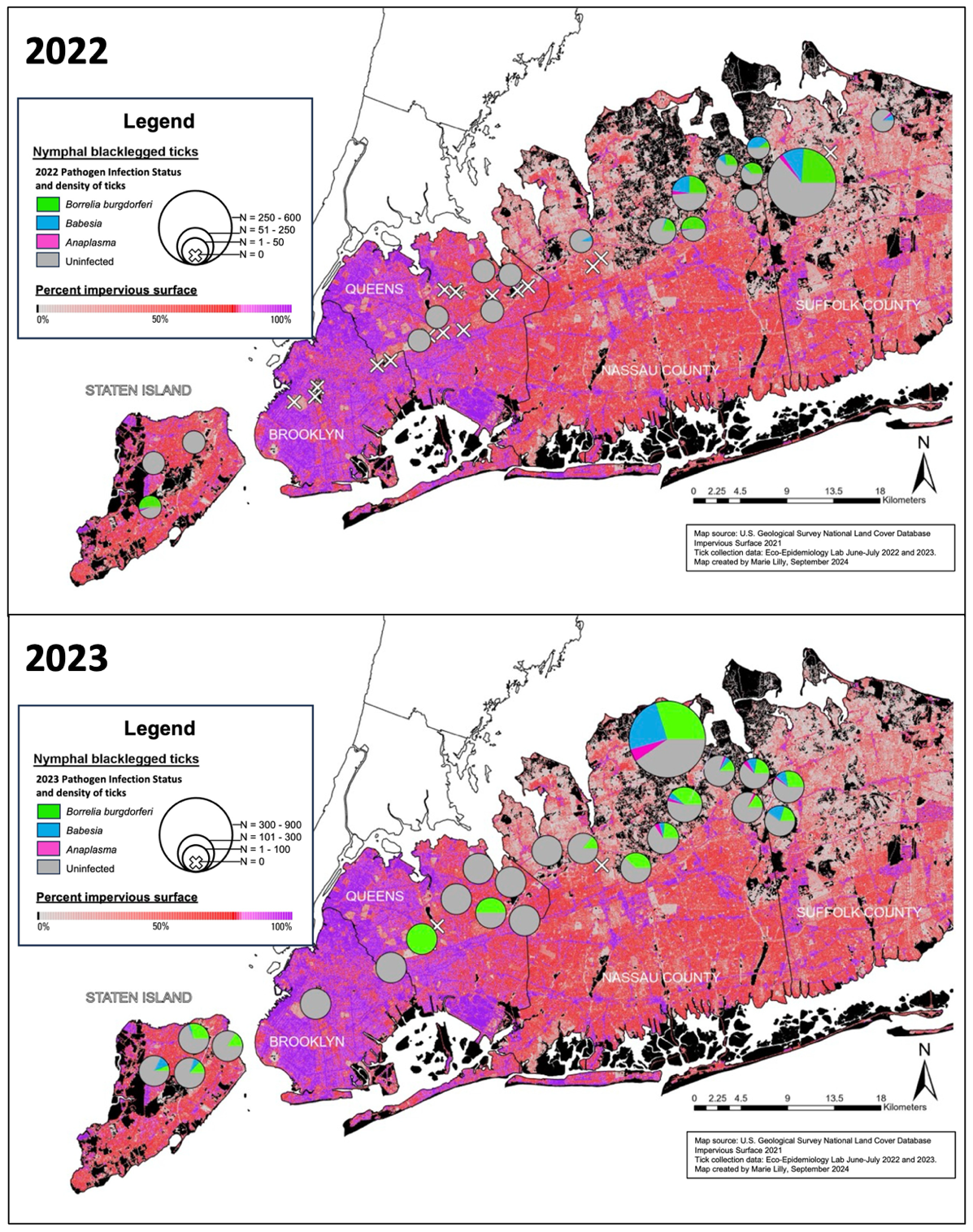


**SI Figure 6.** Maps showing 2022 and 2023 nymphal blacklegged tick collections and infection status with *Borrelia burgdorferi, Babesia microti,* and *Anaplasma phagocytophilum* over percent impervious surface.


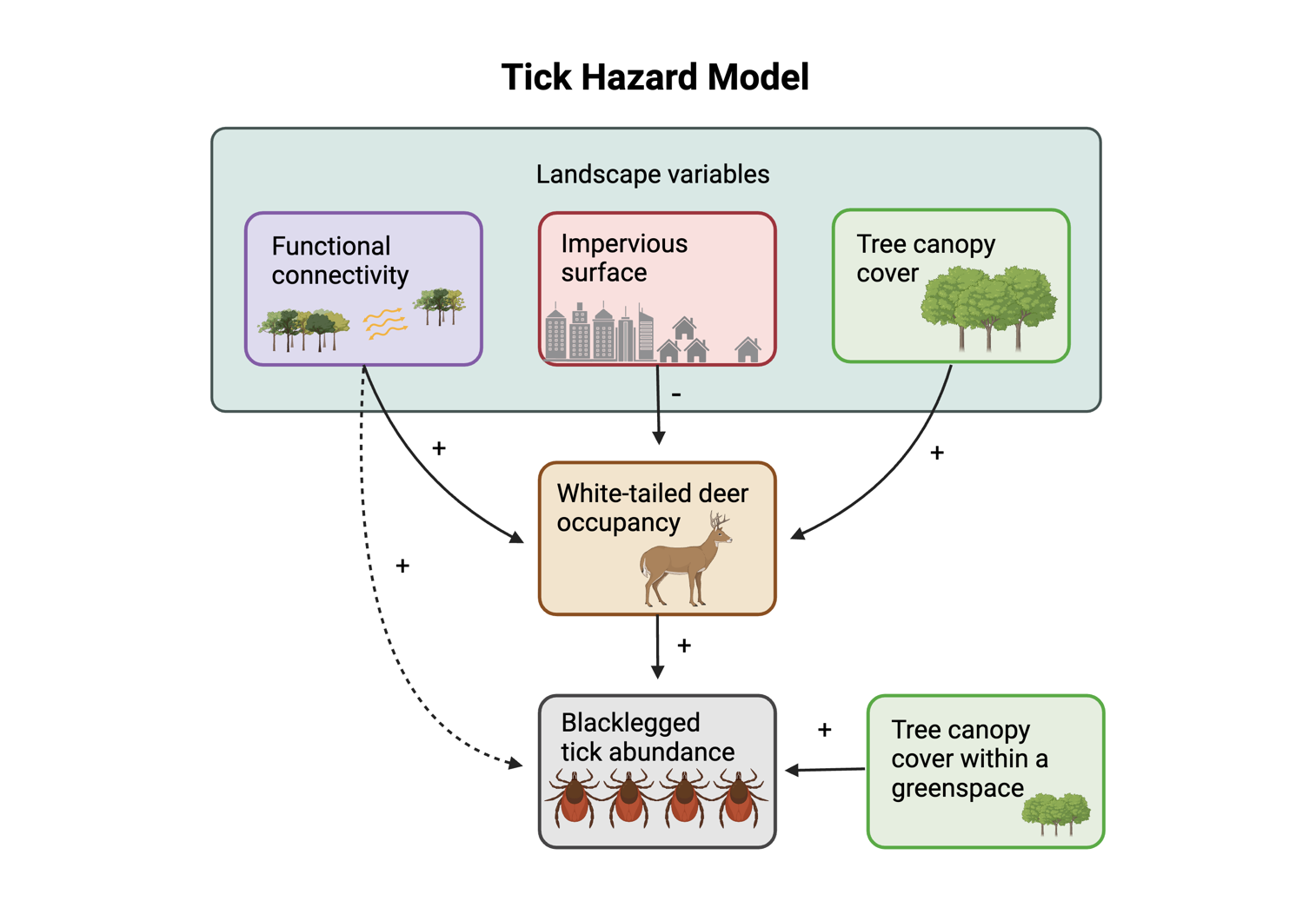


**SI Figure 7.** Summary of relationships found between landscape variables, key tick host (white-tailed deer) occupancy, and blacklegged tick abundance. Direct relationships are indicated with solid lines and the indirect relationship of functional connectivity on blacklegged abundance (via other host movement) is indicated with a dashed line.

**SI Table 1.** Descriptions of landscape variables, land cover layers, and data sources used for landscape analyses and predictors of different response variables assessed.

| **LANDSCAPE DATA LAYERS** | | | | | |
| --- | --- | --- | --- | --- | --- |
| **Response variables** | **Predictor variables** | **Data source** | **Description** | **Justification for use** | **References** |
| Deer occupancy | % Water in buffer | National Land Cover Database (NLCD): Land cover types are derived from Landsat satellite imagery and classified at 30 m x 30 m resolution (Dewitz and U.S. Geological Survey 2021) | "Open Water," "Woody Wetlands," and "Emergent Herbaceous Wetlands" land cover classes were combined to represent available water sources to deer. The percent of available water sources was then calculated within multiple buffer distances around each park polygon using arcGIS. | Open water has been found to limit deer movement, but smaller water bodies and wetlands may positively contribute to urban deer habitat selection. | Coulon et al. 2006, VanAcker et al. 2023 |
| Deer occupancy | Road density | 2023 U.S. Census Bureau Department of Commerce TIGER shapefiles (U.S. Census bureau 2023b) | Total road length was measured within multiple buffer distances around each park polygon using arcGIS. TIGER/Line Shapefile, 2023, County Based Dataset, All Roads. Road density was calculated by summing the total road length within each buffer and dividing it by the buffer area. | Road density can be used as a proxy for deer-vehicle collision potential, which has been found to limit deer movement and occupancy. | Magle et al. 2014, Etter et al. 2002, VanAcker et al. 2019, Girardet et al. 2015 |
| Deer occupancy | Human detection frequency | New York City Urban Wildlife Information Network (UWIN) camera trap data | Human detection frequency was calculated by summing the number of days in the four-month study period with photographs of people in an image. | Human detection has been shown to influence urban wildlife occupancy. | Magle et al. 2014, Lilly Davis et al. in prep |
| Deer detection | %Tree canopy within 100 m buffer of camera trap coordinates | Tree Canopy Cover (TCC): Derived from multi-spectral satellite imagery by the USDA Forest Service (Dewitz 2023) | Percent tree canopy was measured within a 100m buffer of each camera trap coordinate. Percent tree canopy is estimated as a continuous variable across all land cover types | Fine scale tree canopy cover has been found to contribute to differences in detection rates of wildlife camera traps. | Fidino et al. 2020 |
| Deer occupancy, nymphal tick abundance, nymphal tick pathogen infection | Functional connectivity | Derived from the National Land Cover Database (NLCD): Land cover types are derived from Landsat satellite imagery and classified at 30 m x 30 m resolution (Dewitz and U.S. Geological Survey 2021), Omniscape documentation from McRae et al. 2016 | Functional connectivity was modeled using the Omniscape algorithm in Julia package Omniscape.jl | Functional connectivity to deer movement has been positively associated with blacklegged tick abundance in NYC. | Diuk-Wasser et al. 2021, VanAcker et al. 2019 |
| Deer occupancy, nymphal tick abundance, nymphal tick pathogen infection | % Tree canopy within park | Tree Canopy Cover (TCC): Derived from multi-spectral satellite imagery by the USDA Forest Service (Dewitz 2023) | Percent tree canopy was measured within each park polygon using arcGIS. Percent tree canopy is estimated as a continuous variable across all land cover types. | Tree canopy cover within a greenspace has been positively associated with blacklegged tick abundance in NYC | VanAcker et al. 2019 |
| Deer occupancy, nymphal tick abundance, nymphal tick pathogen infection | % Tree canopy in buffer | Tree Canopy Cover (TCC): Derived from multi-spectral satellite imagery by the USDA Forest Service (Dewitz 2023) | Percent tree canopy was measured within multiple buffer distances around each park polygon using arcGIS. Percent tree canopy is estimated as a continuous variable across all land cover types | Tree canopy cover around a greenspace has been positively associated with blacklegged tick abundance in NYC | VanAcker et al. 2019 |
| Deer occupancy, nymphal tick abundance, nymphal tick pathogen infection | % Impervious surface in buffer | National Land Cover Database (NLCD) imperviousness (Dewitz 2023) | Percent impervious surface was measured within multiple buffer distances around each park polygon using arcGIS. Urban impervious surface is estimated as a percentage of developed surface over every 30m pixel | Impervious surface has been found to be negatively associated with blacklegged tick abundance in NYC | VanAcker et al. 2019 |
| Deer occupancy, nymphal tick abundance, nymphal tick pathogen infection | Housing density | 2020 U.S. Census Bureau census tract TIGER shapefiles (U.S. Census bureau 2023a) | Housing density was measured within multiple buffer distances around each park polygon using arcGIS. This dataset identifies the number of housing units per census tract which was then normalized based on the land area of the census tract. | Housing density can be used as a measurement of human dwellings. There is some evidence that deer avoid human dwellings in urban and exurban contexts. | Fidino et al. 2020, Storm et al. 2007 |
| Deer occupancy, nymphal tick abundance, nymphal tick pathogen infection | Patch size | Protected Area Database of the US (PAD-US) (USGS 2020), New York State parks (NYS Parks Administration 2012), New York City Parks (NYC Parks Open Data Team 2024) | Shapefiles for open green spaces were used in arcGIS to measure the area of each open greenspace as a "patch" dictated by the political boundary of the greenspace. Patch size was calculated by hand drawing features in arcGIS for sites where a pre-existing shapefile was not available. | Urban patch size may influence host (i.e. deer) occupancy and has been the main landscape metric used as a predictor of tick abundance. | Magle et al. 2014, LoGiudice et al. 2008, Allan et al. 2003) |
| Nymphal tick abundance, nymphal tick pathogen infection | % Bare soil in buffer | National Land Cover Database (NLCD): Derived from Landsat satellite imagery land cover types are classified at 30 m x 30 m resolution (Dewitz and U.S. Geological Survey 2021) | "Barren Land” land cover class used to represent bare soil and other accumulations of earthen material. Generally, vegetation accounts for less than 15% of total cover for each pixel classified as "Barren Land." The percent available water sources was then calculated within multiple buffer distances around each park polygon using arcGIS. | Bare soil around greenspaces was found negatively associated with blacklegged nymphal tick abundance in NYC | VanAcker et al. 2019 |


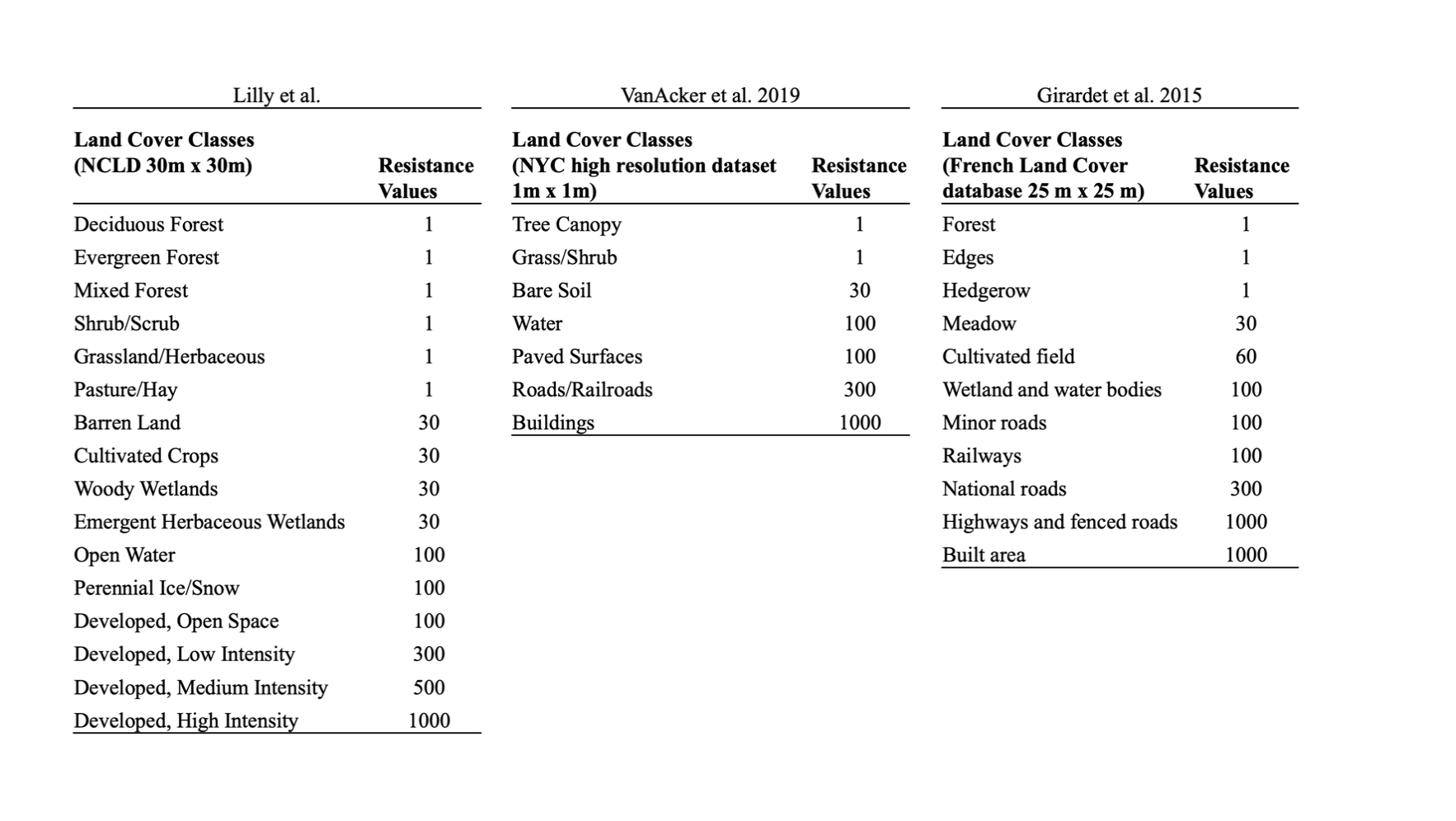
**SI Table 2.** Resistance values adapted from VanAcker et al. 2019 and Girardet et al. 2015 to fit National Land Cover Database (NLCD) Land use land cover (Dewitz and U.S. Geological Survey 2021)

**SI Table 3.** *Ixodes scapularis* nymphal pathogen infection prevalence across 38 sites in five counties from New York City through Long Island, NY (2022-2023).

|  |  | **YEAR 2022** | | | | | **YEAR 2023** | | | | |
| --- | --- | --- | --- | --- | --- | --- | --- | --- | --- | --- | --- |
| **SITE** | **COUNTY** | ***NIP Borrelia burgdorferi*** | **NIP *Babesia microti*** | **NIP *Anaplasma phagocytophilum*** | ***Ixodes scapularis* nymphs (n)** | **Tested (n)** | ***NIP Borrelia burgdorferi*** | **NIP *Babesia microti*** | **NIP *Anaplasma phagocytophilum*** | ***Ixodes scapularis* nymphs (n)** | **Tested (n)** |
| Brookfield | Staten Island | 0.52 | 0.02 | 0.02 | 48 | 48 | NA | NA | NA | NA | NA |
| Clove Lakes | Staten Island | 0.00 | 0.00 | 0.00 | 5 | 3 | 0.30 | 0.02 | 0.00 | 62 | 50 |
| Deere | Staten Island | NA | NA | NA | NA | NA | 0.16 | 0.00 | 0.00 | 54 | 50 |
| Todt Hill | Staten Island | NA | NA | NA | NA | NA | 0.13 | 0.07 | 0.00 | 15 | 15 |
| Willowbrook | Staten Island | 0.00 | 0.00 | 0.00 | 7 | 8 | 0.06 | 0.13 | 0.00 | 16 | 16 |
| The Green-Wood Cemetery | Brooklyn | NA | NA | NA | 0 | 0 | NA | NA | NA | NA | NA |
| Prospect I | Brooklyn | NA | NA | NA | 0 | 0 | 0.00 | 0.00 | 0.00 | 3 | 3 |
| Prospect II | Brooklyn | NA | NA | NA | 0 | 0 | NA | NA | NA | NA | NA |
| The Evergreens Cemetery | Brooklyn | NA | NA | NA | 0 | 0 | NA | NA | NA | NA | NA |
| Alley Pond | Queens | NA | NA | NA | 0 | 0 | 0.00 | 0.00 | 0.00 | 3 | 3 |
| Captain Tilly | Queens | NA | NA | NA | 0 | 0 | NA | NA | NA | NA | NA |
| Cunningham I | Queens | 0.00 | 0.00 | 0.00 | 1 | 0 | NA | NA | NA | 0 | 0 |
| Cunningham II | Queens | NA | NA | NA | 0 | 0 | 1.00 | 0.00 | 0.00 | 1 | 1 |
| Flushing Meadows | Queens | 0.00 | 0.00 | 0.00 | 1 | 0 | NA | NA | NA | 0 | 0 |
| Forest I | Queens | 0.00 | 0.00 | 0.00 | 1 | 0 | 1.00 | 0.00 | 0.00 | 1 | 1 |
| Forest II | Queens | NA | NA | NA | 0 | 0 | NA | NA | NA | 0 | 0 |
| Highland | Queens | NA | NA | NA | 0 | 0 | 0.00 | 0.00 | 0.00 | 2 | 2 |
| Horatio Playground | Queens | 0.00 | 0.00 | 0.00 | 2 | 3 | 0.00 | 0.00 | 0.00 | 5 | 2 |
| Kissena Corridor | Queens | NA | NA | NA | 0 | 0 | NA | NA | NA | NA | NA |
| Kissena | Queens | NA | NA | NA | 0 | 0 | 0.00 | 0.00 | 0.00 | 1 | 1 |
| Maplegrove Cemetery | Queens | NA | NA | NA | 0 | 0 | NA | NA | NA | NA | NA |
| North Alley Pond | Queens | 0.00 | 0.00 | 0.00 | 2 | 2 | 0.00 | 0 | 0 | 3 | 3 |
| Queens County Farm Museum | Queens | NA | NA | NA | 0 | 0 | NA | NA | NA | NA | NA |
| Brookville Nature | Nassau | 0.51 | 0.01 | 0.01 | 67 | 67 | 0.40 | 0.06 | 0.03 | 237 | 100 |
| Christopher Morley | Nassau | 0.00 | 0.07 | 0.00 | 14 | 14 | 0.14 | 0.00 | 0.00 | 14 | 14 |
| Clark Botanic Gardens | Nassau | NA | NA | NA | 0 | 0 | NA | NA | NA | 0 | 0 |
| Fox Hollow | Nassau | 0.28 | 0.12 | 0.00 | 25 | 25 | 0.12 | 0.06 | 0.02 | 163 | 100 |
| John D. Caemmerer | Nassau | NA | NA | NA | 0 | 0 | NA | NA | NA | NA | NA |
| Muttontown | Nassau | 0.29 | 0.27 | 0.04 | 165 | 100 | 0.34 | 0.28 | 0.06 | 957 | 100 |
| Old Westbury Gardens | Nassau | NA | NA | NA | NA | NA | 0.38 | 0.00 | 0.00 | 91 | 50 |
| Stillwell Woods | Nassau | 0.38 | 0.03 | 0.00 | 29 | 29 | 0.24 | 0.12 | 0.05 | 139 | 100 |
| SUNY Old Westbury | Nassau | 0.19 | 0.02 | 0.00 | 98 | 98 | 0.24 | 0.06 | 0.05 | 168 | 100 |
| Trail View SP | Nassau | 0.00 | 0.00 | 0.00 | 8 | 13 | 0.16 | 0.00 | 0.03 | 39 | 38 |
| Whitney Pond | Nassau | 0.00 | 0.00 | 0.00 | 2 | 0 | 0.00 | 0.00 | 0.00 | 5 | 5 |
| Breezy | Suffolk | 0.11 | 0.44 | 0.00 | 9 | 9 | 0.25 | 0.25 | 0.00 | 10 | 4 |
| Frazer Drive | Suffolk | NA | NA | NA | 0 | 0 | NA | NA | NA | NA | NA |
| Froehlich Farm Nature Preserve | Suffolk | 0.25 | 0.11 | 0.02 | 564 | 100 | 0.30 | 0.12 | 0.02 | 164 | 50 |
| Veterans | Suffolk | 0.00 | 0.09 | 0.03 | 32 | 32 | 0.15 | 0.11 | 0.1 | 188 | 100 |
|  | **OVERALL** | **0.26** | **0.11** | **0.02** | **1080** | **551** | **0.27** | **0.15** | **0.04** | **2341** | **908** |

**SI Table 4.** Deer detection models (*p*) with null occupancy model (*Ψ* (.)) nPars represents the number of parameters in the model.

| **Model** | **nPars** | **AIC** | **ΔAIC** | **AIC weight** | **Cumulative weight** |
| --- | --- | --- | --- | --- | --- |
| *Ψ*(.)*p*(TCC + Season) | 6 | 366.62 | 0 | 0.93 | 0.93 |
| *Ψ*(.)*p*(TCC) | 3 | 372.05 | 5.43 | 0.06 | 1 |
| *Ψ*(.)*p*(Season) | 5 | 378.05 | 11.43 | <0.01 | 1 |
| *Ψ*(.)*p*(.) | 2 | 383.14 | 16.52 | <0.01 | 1 |

**SI Table 5.** Best-fit model for probability of detection (*p*) of deer with all seasons compared to one another.

| **Model component** | **Estimate** | **SE** | **z** | **P(>\|z\|)** |
| --- | --- | --- | --- | --- |
| (Intercept) | -0.73 | 0.28 | -2.62 | 0.009 |
| TCC | 0.76 | 0.22 | 3.51 | <0.001 |
| Fall (compared to Winter) | -1.03 | 0.38 | -2.69 | 0.007 |
| Spring (compared to Winter) | -1.27 | 0.46 | -2.78 | 0.005 |
| Summer (compared to Winter) | -0.93 | 0.37 | -2.50 | 0.013 |
| Spring (compared to Fall) | -0.24 | 0.45 | -0.53 | 0.593 |
| Summer (compared to Fall) | 0.10 | 0.36 | 0.27 | 0.787 |
| Winter (compared to Fall) | 1.03 | 0.38 | 2.69 | 0.007 |
| Fall (compared to Summer) | -0.10 | 0.36 | -0.27 | 0.787 |
| Spring (compared to Summer) | -0.34 | 0.44 | -0.77 | 0.442 |
| Winter (compared to Summer) | 0.93 | 0.37 | 2.50 | 0.013 |
| Fall (compared to Spring) | 0.24 | 0.45 | 0.53 | 0.593 |
| Summer compared to Spring) | 0.34 | 0.44 | 0.77 | 0.442 |
| Winter (compared to Spring) | 1.27 | 0.46 | 2.78 | 0.005 |

TCC=Percent tree canopy cover within 100m of camera trap

**SI Table 6.** Deer occupancy models (*Ψ*). The null model (detection only) is listed for comparison. nPars represents the number of parameters in the model.

| **Model** | **nPars** | **AIC** | **ΔAIC** | **AIC weight** | **Cumulative weight** |
| --- | --- | --- | --- | --- | --- |
| *Ψ* (TCC 1000 m greenspace buffer)*p*(TCC + Season) | 7 | 320.81 | 0 | 3.50 | 0.35 |
| *Ψ* (Impervious surface 1000 m greenspace buffer)*p*(TCC + Season) | 7 | 320.97 | 0.15 | 3.30 | 0.68 |
| *Ψ* (Connectivity 1000 m greenspace buffer)*p*(TCC + Season) | 7 | 320.97 | 0.16 | 3.20 | 1 |
| *Ψ* (.)*p*(TCC + Season) | 2 | 383.14 | 62.33 | <0.001 | 1 |

TCC = Percent tree canopy cover

**SI Table 7.** Nymphal blacklegged tick abundance as a function of candidate deer occupancy model predictions. nPars represents the number of parameters in the model.

| **Occupancy Model** | **nPars** | **AIC** | **ΔAIC** | **AIC weight** | **Cumulative weight** |
| --- | --- | --- | --- | --- | --- |
| Functional connectivity | 5 | 405.88 | 0 | 0.96 | 0.96 |
| Model averaged: Functional connectivity, Impervious surface, TCC | 6 | 413.08 | 7.19 | 0.03 | 0.99 |
| Percent impervious surface | 5 | 415.90 | 10.02 | 0.01 | 1 |
| Percent tree canopy cover (TCC) | 5 | 416.70 | 10.82 | 0 | 1 |

**SI Formula 1.** Single-season occupancy model

The single-season occupancy model used can be written as follows:

*y_i_|z_i_* ∼ Bernoulli(*p_i_ ∗ z_i_*)

*z_i_* ∼ Bernoulli(*Ψ_i_*)

Logit (*p_i_*) = α * Detection Covariate_i_

Logit (*Ψ_i_*) = *β ** Occupancy Covariate_i_

Where,

*y* = the binary species detection data at site

*p =* detection probability

*z =* true occupancy state

*i =* site

*Ψ* = occupancy probability

α = the parameters for estimating detection probability

*β =* parameters for estimating occupancy probability

**Section S1.**

**METHODS**

*Tick hazard NIP models*

It is difficult to accurately determine nymphal pathogen infection prevalence with low sample sizes, and this problem can introduce bias into infection prevalence estimations (Jovani and Tella 2006). Across all sites and years, we had 44/63 sampling points with fewer than 25 nymphal *I. scapularis* ticks collected. While there are different minimum sample sizes suggested in the literature (VanAcker et al. 2019, Diuk-Wasser et al. 2012, Horobik et al. 2007, Foster et al. 2023), the CDC recommends using a threshold of 25 ticks for determining nymphal infection prevalence (CDC 2024).

We constructed binomial family Generalized Linear Mixed Models (GLMMs) weighted by sample size with site and year as random effects to evaluate deer occupancy as a predictor of nymphal infection prevalence with 1) *Borrelia burgdorferi* 2) *Babesia microti*, and 3) *Anaplasma phagocytophilum*. We compared the full dataset (64 observations across all sites and years), the dataset with a minimum of 10 ticks tested (24 observations across all sites and years), and the dataset with a minimum of 25 ticks tested (19 observations across all sites and years).

**RESULTS**

*Tick hazard NIP*

We found that deer occupancy was significantly positively associated (estimate = 1.05, p = 0.04) with nymphal infection prevalence with *B. burgdorferi* when the full dataset was included in the model. The positive association between deer occupancy and nymphal infection prevalence with *B. burgdorferi* was still observed when the data was subset to include a minimum of 10 ticks tested and a minimum of 25 ticks tested. However, this relationship was no longer significant. Deer occupancy was positively associated with both nymphal infection prevalence with *B. microti* and *A. phagocytophilum* in all models constructed, but this relationship was not significant (SI. Table 8).

**SI Table 8.** Nymphal infection prevalence (NIP) with *Borrelia burgdorferi*, *Babesia microti*, and *Anaplasma phagocytophilum* as a function of white-tailed deer occupancy

| **Response variable** | **Model component** | **Estimate** | **SE** | **z value** | **Pr(>\|z\|)** |
| --- | --- | --- | --- | --- | --- |
| NIP *B. burgdorferi,*  full dataset | (Intercept) | -2.22 | 0.47 | -4.76 | <0.001 |
|  | White-tailed deer occupancy | 1.05 | 0.52 | 2.03 | 0.04 |
| NIP *B. burgdorferi,*  ≥10 ticks | (Intercept) | -1.69 | 0.55 | -3.05 | <0.001 |
|  | White-tailed deer occupancy | 0.56 | 0.61 | 0.93 | 0.35 |
| NIP *B. burgdorferi,*  ≥25 ticks | (Intercept) | -1.25 | 0.58 | -2.18 | 0.03 |
|  | White-tailed deer occupancy | 0.24 | 0.62 | 0.39 | 0.70 |
| *NIP B. microti,*  full dataset | (Intercept) | -4.38 | 0.90 | -4.87 | <0.001 |
|  | White-tailed deer occupancy | 1.66 | 0.98 | 1.70 | 0.09 |
| *NIP B. microti,*  ≥10 ticks | (Intercept) | -3.51 | 0.98 | -3.59 | <0.001 |
|  | White-tailed deer occupancy | 0.63 | 1.07 | 0.59 | 0.56 |
| *NIP B. microti,*  ≥25 ticks | (Intercept) | -4.66 | 1.41 | -3.32 | <0.001 |
|  | White-tailed deer occupancy | 1.82 | 1.48 | 1.23 | 0.22 |
| *NIP A. phagocytophilum,* full dataset | (Intercept) | -6.91 | 2.09 | -3.30 | <0.001 |
|  | White-tailed deer occupancy | 3.19 | 2.09 | 1.52 | 0.13 |
| *NIP A. phagocytophilum,* ≥10 ticks | (Intercept) | -6.54 | 2.10 | -3.11 | 0.002 |
|  | White-tailed deer occupancy | 2.86 | 2.11 | 1.36 | 0.17 |
| *NIP A. phagocytophilum,* ≥25 ticks | (Intercept) | -6.08 | 1.99 | -3.06 | 0.002 |
|  | White-tailed deer occupancy | 2.48 | 2.00 | 1.24 | 0.21 |

**DISCUSSION**

Because we found low nymphal tick densities at many of our most urban sites, excluding sites with low tick sample sizes from our nymphal infection prevalence models skews the results towards sites where deer occupancy is less heterogeneous. However, we are not confident in nymphal infection prevalence estimates for sites with low tick sample sizes. Thus, we conclude that the density of infected nymphs is more accurate than infection prevalence for understanding how deer occupancy influences pathogen infection across an urbanization gradient.

**SI Additional References**

CDC. 2024. “Surveillance for *Ixodes scapularis* and pathogens found in this tick species in the United States.” https://www.cdc.gov/ticks/resources/TickSurveillance_Iscapularis-P.pdf

Coulon, A., Guillot, G., Cosson, J.-F., Angibault, J. M. A., Aulagnier, S., Cargnelutti, B., Galan, M., & Hewison, A. J. M. (2006). Genetic structure is influenced by landscape features: Empirical evidence from a roe deer population. *Molecular Ecology*, *15*(6), 1669–1679. <https://doi.org/10.1111/j.1365-294X.2006.02861.x>

Diuk-Wasser, M. A., Hoen, A. G., Cislo, P., Brinkerhoff, R., Hamer, S. A., Rowland, M., Cortinas, R., Vourc’h, G., Melton, F., Hickling, G. J., Tsao, J. I., Bunikis, J., Barbour, A. G., Kitron, U., Piesman, J., & Fish, D. (2012). Human Risk of Infection with Borrelia burgdorferi, the Lyme Disease Agent, in Eastern United States. *The American Journal of Tropical Medicine and Hygiene*, *86*(2), 320–327.<https://doi.org/10.4269/ajtmh.2012.11-0395>

Foster, E., Maes, S. A., Holcomb, K. M., & Eisen, R. J. (2023). Prevalence of five human pathogens in host-seeking *Ixodes scapularis* and *Ixodes pacificus* by region, state, and county in the contiguous United States generated through national tick surveillance. *Ticks and Tick-Borne Diseases*, *14*(6), 102250.<https://doi.org/10.1016/j.ttbdis.2023.102250>

Horobik, V., Keesing, F., & Ostfeld, R. S. (2007). Abundance and Borrelia burgdorferi-infection Prevalence of Nymphal Ixodes scapularis Ticks along Forest–Field Edges. *EcoHealth*, *3*(4), 262–268.<https://doi.org/10.1007/s10393-006-0065-1>

Jovani, R., & Tella, J. L. (2006). Parasite prevalence and sample size: Misconceptions and solutions. *Trends in Parasitology*, *22*(5), 214–218.<https://doi.org/10.1016/j.pt.2006.02.011>

Storm, D. J., Nielsen, C. K., Schauber, E. M., & Woolf, A. (2007). Space Use and Survival of White-Tailed Deer in an Exurban Landscape. *The Journal of Wildlife Management*, *71*(4), 1170–1176. <https://doi.org/10.2193/2006-388>

VanAcker, M. C., Little, E. A. H., Molaei, G., Bajwa, W. I., & Diuk-Wasser, M. A. (2019). Enhancement of Risk for Lyme Disease by Landscape Connectivity, New York, New York, USA. *Emerging Infectious Diseases*, *25*(6), 1136–1143. <https://doi.org/10.3201/eid2506.181741>
